# Validation of Splicing Events in Transcriptome Sequencing Data

**DOI:** 10.3390/ijms18061110

**Published:** 2017-05-23

**Authors:** Wolfgang Kaisers, Johannes Ptok, Holger Schwender, Heiner Schaal

**Affiliations:** 1 Department for Anaesthesiology, University Hospital Düsseldorf, Heinrich Heine University, 40225 Düsseldorf, Germany; 2 BMFZ (Biologisch-Medizinisches Forschungszentrum), Heinrich Heine University, 40225 Düsseldorf, Germany; schwender@math.uni-duesseldorf.de (H.S.); schaal@uni-duesseldorf.de (H.S.); 3 Institute of Virology, University Hospital Düsseldorf, Heinrich Heine University, 40225 Düsseldorf, Germany; Johannes.Ptok@uni-duesseldorf.de; 4 Mathematical Institute, Heinrich Heine University, 40225 Düsseldorf, Germany

**Keywords:** splice sites, RNA-seq, TopHat, STAR, MaxEnt

## Abstract

Genomic alignments of sequenced cellular messenger RNA contain gapped alignments which are interpreted as consequence of intron removal. The resulting gap-sites, genomic locations of alignment gaps, are landmarks representing potential splice-sites. As alignment algorithms report gap-sites with a considerable false discovery rate, validations are required. We describe two quality scores, gap quality score (*gqs*) and weighted gap information score (*wgis*), developed for validation of putative splicing events: While *gqs* solely relies on alignment data *wgis* additionally considers information from the genomic sequence. FASTQ files obtained from 54 human dermal fibroblast samples were aligned against the human genome (GRCh38) using TopHat and STAR aligner. Statistical properties of gap-sites validated by *gqs* and *wgis* were evaluated by their sequence similarity to known exon-intron borders. Within the 54 samples, TopHat identifies 1,000,380 and STAR reports 6,487,577 gap-sites. Due to the lack of strand information, however, the percentage of identified GT-AG gap-sites is rather low. While gap-sites from TopHat contain ≈89% GT-AG, gap-sites from STAR only contain ≈42% GT-AG dinucleotide pairs in merged data from 54 fibroblast samples. Validation with *gqs* yields 156,251 gap-sites from TopHat alignments and 166,294 from STAR alignments. Validation with *wgis* yields 770,327 gap-sites from TopHat alignments and 1,065,596 from STAR alignments. Both alignment algorithms, TopHat and STAR, report gap-sites with considerable false discovery rate, which can drastically be reduced by validation with *gqs* and *wgis*.

## 1. Introduction

Analysis of transcriptome sequencing data focuses on differential expression of genes, as well as alternative splicing. Genomic alignments of transcriptome sequencing data contain alignment gaps. Alignment gaps are landmarks indicating potential splice-sites. Thus, due to the complexity of eukaryotic transcriptomes, transcript reconstruction from sequenced mRNA encompasses considerable ambiguities. Low specificity of reported alignment gaps may seriously compromise validity of analysis results. Currently, transcript reconstruction algorithms suffer from inaccuracies [1,2] and even the (much simpler) identification of splice events is associated with a high false discovery rate (FDR) [3]. Additionally, lack of strand information makes assignment of the correct strand difficult.

The FDR can be reduced by additional validations. Early and simplifying “validation” procedures include restriction to (for example) GT-AG-sites [4] and filtering for minimal exonic match length or filtering for minimal number of supporting alignments (as reported by RGASP, RNA-seq Genome Annotation Assessment Project [3]).

Recently 184 splice sites with non-canonical dinucleotides and U2/U12 like consensus sequences have been reported [5] and therefore, filter based on intronic dinucleotides are likely to be inappropriate. The rationale for usage of quality scores is to differentiate between biology (for example regulated splicing events) and artefacts (for example stochastic noise in splicing, sequencing and alignment). In the following, two approaches for validation of splicing events in transcriptome sequencing data are described and evaluated: An approach solely relying on alignment data (*gqs*) and a second approach which additionally includes information from genomic sequence (*wgis*).

### 1.1. Genomic Alignments and Gap-Sites

**Figure 1:**
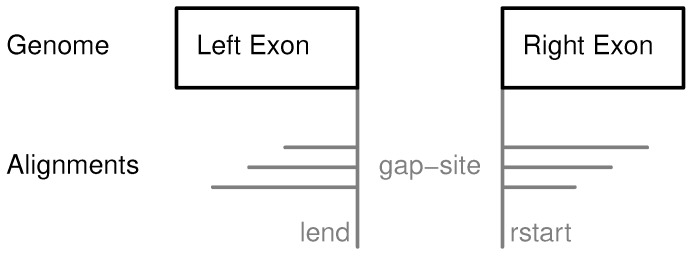
Definition of gap-sites. Since no strand information is provided by alignments, gap-site positions are defined reading direction of genomic sequence (left to right). *lend* is defined as position of last exonic nucleotide on the left side. *rstart* is defined as position of first exonic nucleotide on right side. The “left” and “right” side are directions on genomic reference sequence, defined by lower and higher position coordinates respectively relative to the actual reading position (in accordance with common reading orientation). The gap-site is covered by three alignments (nAligns = 3).

The following section describes the definition of gap-sites and the parameters collected from alignment data. Alignment results are reported in BAM files and structure of genomic alignments is described in BAM file format [6] (see also Appendix A.1). Alignment data contained in BAM files (currently) does not include strand information because it is lost during sequencing [7]. In genomic alignments of sequenced cellular mRNA, reads crossing splice sites cover at least two exons which are separated by an intron and thus result in gapped alignments (Figure 1). Alignment gap locations possibly shared by multiple alignments define a gap-site. Assuming that alignment gaps result from splicing events, gap-site positions are candidates for splice junctions. A gap-site is characterised by two genomic positions: *lend* and *rstart* and the alignments are called “supporting alignments”. The number of supporting alignments is called alignment coverage or nAligns-value for the respective gap-site. The number of samples in which a gap-site is identified is called the multiplicity (nProbes) value for this gap-site.

For single alignments covering gap-sites, the minimum length of both (exonic) nucleotide matching regions (the minimum CIGAR length value or *mcl* value, described in Appendix A.1.3) is included into *gqs*. The *mcl* criterion provides a lower limit for support of an alignment gap by subsequent nucleotide matches. Two other alignment derived values, quartet sum of *mcl* (=*qsm*) and number of distinct (left) alignment start positions (*nlstart*) are described in detail in Sections Appendix A.1.4 and Appendix A.1.5 respectively.

On the plus-strand the splice-site donor (or 5${}^{\prime }$ splice-site) is located at *lend* position and the splice-site acceptor (or 3${}^{\prime }$ splice-site) is located at *rstart* position (Figure 1). On the minus-strand the opposite rule applies.

#### 1.1.1. Intronic Genome Sequence at Gap-Sites

Statistical properties of gap-sites are evaluated using distribution of intronic dinucleotides (IDIN, for example GT) and IDIN-pairs (for example GT-AG) and sequence logos. Therefore, the two subsequent nucleotides following the *lend* position and preceding the *rstart* position are analysed.

The location of IDIN is called “left” or “right” as long as no strand information is considered or available. Using strand information exon-intron boundaries can further be classified into 5${}^{\prime }$- and 3${}^{\prime }$-splice-sites. For “+”-strand, the raw left IDIN are from 5${}^{\prime }$ splice-junctions (called 5${}^{\prime }$IDIN) and the raw right IDIN are from 3${}^{\prime }$ splice-sites (called 3${}^{\prime }$IDIN). For “−”-strand, the reverse-complement of right IDIN are from 5${}^{\prime }$ splice-junctions (called 5${}^{\prime }$IDIN) and the reverse-complement of left IDIN are from 3${}^{\prime }$ splice-junctions (called 3${}^{\prime }$IDIN).

Gap-sites are referred to by 5${}^{\prime }$IDIN (for example GT-sites) or by IDIN-pairs (for example GT-AG-sites or AT-AC-sites). From here on, IDIN and IDIN-pairs in strand-corrected orientation (5${}^{\prime }$ to 3${}^{\prime }$) are shown in italic letters. Non cursive printed nucleotides represent uncorrected genomic sequence (left to right).

#### 1.1.2. General Considerations on Quality Scores

Recognition of rare events (for example non-canonical splicing) requires high sensitivity and therefore data is merged from multiple samples and large amounts of alignment data. Merging of data from multiple samples and the structure of BAM files implicate that validating information on gap-sites emerges sequentially. Therefrom, two basic requirements for gap-site scores derive:

- Monotonicity: By adding new alignment information, the score must not decrease and should increase with the number of alignment matches to genomic nucleotides. Due to monotonicity, mean or median values cannot be used. This criterion precludes that an initially high score decreases when low scoring alignments are added.
- Informational limit: Collection of information for each gap-site is restricted to a static size where splicing events are sufficiently confirmed. Using this limit, data from multiple alignments can be packed into integer variables allowing fast processing without necessity of specialised data containers.

### 1.2. Definition of Gap Quality Score (gqs)

The Gap Quality Score (*gqs*) solely uses information extracted from genomic alignments and therefore can directly be calculated from (multiple) BAM files. As shown in Equation (1), *gqs* essentially consists of two factors: *qsm* and *nlstart*. A calculated example is given in Appendix (Table A1). The *gqs* is distributed between 0 and 1000 (for read length of 100 and on a 64 bit operating system). A score of 1000 requires at least 8 supporting alignments and four maximal *mcl* values (at least 50 matching nucleotides on both sides in at least 4 alignment gaps for *qsm*= 200). Gap-sites attaining a *gqs* of 1000 are called “*gqs*-validated” (see Figure 6b for empirical base of definition).

The structure of *gqs* implies that the probability of being *gqs*-validated increases with higher alignment coverage.

The *gqs* is defined as:
  (1) $$gqs=10\times \frac{\mathrm{nlstart}}{n}\;\frac{2\times \mathrm{qsm}}{4}.$$
The number of different alignment start positions for a gap-site denoted *nlstart* value (see Appendix A.1.5). *qsm* is the sum of the four largest minor alignment match lengths beside the alignment gap (see Appendix A.1.4). *n* is the number of bytes in an integer (n=4 on a 32 bit system and n=8 on a 64 bit operating system). The gqs additionally is truncated to integral values.

### 1.3. Definition of Weighted Gap Information Score (wgis)

The Weighted Gap Information Score (*wgis*) is a successor of *gqs* because of the *gqs* being too insensitive on gap-sites supported by only few alignments. From alignments, the *wgis* basically utilises the same input values as *gqs* (*qsm* and *nlstart*). Additionally, the similarity of the genomic sequence with splice-sites is evaluated using MaxEnt scores: *score5* for 5${}^{\prime }$ splice-sites and *score3* for 3${}^{\prime }$ splice-sites [8,9]. All factors are weighted (using log2). Thresholds are applied to *qsm*, *score5* and *score3*. Information on empirical base for thresholds is provided in supplemental material. For gap-sites with any quality below a given threshold, *wgis* =0 is returned. All sites with *wgis* ≠ 0 are called “*wgis*-validated”. Strand information is reported by *wgis* via signature. The detailed definition of *wgis* is given in Equation (2).

The *wgis* is defined as:

(2) $$wgis=f_{nls}\times f_{qsm}\times f_{s5}\times f_{s3}\times s_{str}.$$

**Factor**
**Name**
**Source**
**Definition**
**Threshold**
**Maximal Value**

$f_{nls}$
*nlstart*-factorBAM-file
$\log_{2}\left(\log_{2}\left(nlstart\right)+1\right)+1$

3
$f_{qsm}$
*qsm*-factorBAM-file
$\log_{2}\left(\log_{2}\left(\max\left(qsm-13,2\right)\right)\right)$
152.916
$f_{s5}$
*score5*-factorMaxEnt
$\log_{2}\left(\max\left(score5,1\right)\right)$
13.56
$f_{s3}$
*score3*-factorMaxEnt
$\log_{2}\left(\max\left(score3,1\right)\right)$
14.01
$s_{str}$
strand-signMaxEnt

1The BAM-file derived factor $f_{nls}$ is positive (>0). The BAM-file derived factor $f_{qsm}$ and the two MaxEnt score factors ($f_{s5}$, $f_{s3}$) are non-negative (≥ 0). Therefore *wgis* = 0 exactly when qsm≤15 or score5≤1 or score3≤1 (threshold). The sign of *wgis* is determined by the strand-sign $sgn\left(wgis\right)=sgn\left(s_{str}\right)$. Maximal observed MaxEnt scores on annotated splice-sites are 11.8 for *score5* and 16.1 for *score3* leading to a theoretical maximum of 124.9 for *wgis* (on a 64 bit operating system).

#### 1.3.1. Sequence Similarity between Gap-Sites and Splice-Sites

The probability of a gap-site being a true splice-site is rated using the MaxEnt score developed by Burge et al. The MaxEnt score uses a log-odd ratio (defined as ratio of sequence motif frequency in true splice sites and decoy). Sequence 9-mers and 23-mers are analysed for evaluation of 5${}^{\prime }$ and 3${}^{\prime }$ splice-sites respectively (positions −3 to +6 for 5${}^{\prime }$ sites and −20 to +3 for 3${}^{\prime }$ sites). The *maximum-entropy* is an estimation procedure for probability distributions originally developed in statistical mechanics [10,11]. In an iterative approach, the maximum entropy distribution has been estimated in advance. Scores are extracted from pre-calculated tables based on sequence related indices. In their paper, Burge et.al. describe that a training data set had been derived from a set of 1821 transcripts spanning 12,715 introns (5${}^{\prime }$ and 3${}^{\prime }$ splice sites).

#### 1.3.2. Strand Information in *wgis*

The strand sign ($s_{str}$) of *wgis* validated gap-sites is determined by evaluation of MaxEnt*score3*. Calculation of *score3* at the left exon-intron boundary (around *lend* position) in original orientation (left to right) yields *score3* in “+”-strand orientation (*score3*(+)). Calculation of *score3* at the right exon-intron boundary (around *rstart* position) in reverse-complement orientation (right to left) yields *score3* in “−”-strand orientation (*score3*(−)). The strand sign is calculated by comparison of these two values as shown in Equation (3).

(3) $$s_{str}=\left\{\begin{array}{ccc} +1 & \mathrm{when} & score3(+)\leq score3(-)\\ -1 & \mathrm{otherwise} & \end{array}\right.$$

The biological interpretation is, that when the right boundary of a gap-site has stronger resemblance to a 3${}^{\prime }$ splice-site than the left boundary of the gap-site (read in reversed direction), then “+”-strand is assumed.

### 1.4. GQL

The distribution of *wgis*, naturally separates gap-sites into four sub-populations. The derived categories consist of quality levels 0 to 3 named “Gap Quality Level” (*gql*): *gql*0 to *gql*3. Gap-sites assigned to *gql*-level i are denoted *gql*i-sites. The set of *gql*0 sites for example is a s “not *wgis*-validated” (wgis=0). Details of *gql* definition are described later on in Section 2.3.1.

### 1.5. Annotation of Gap-Sites

**Figure 2:**
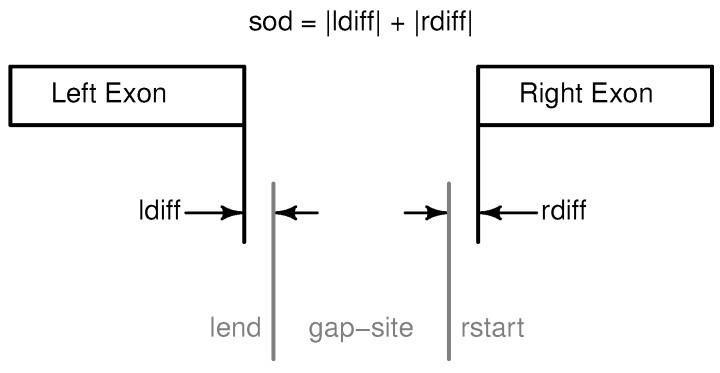
Annotation of gap-sites in R package *refGenome*. Annotation of a gap-sites as implemented in (CRAN) R package *refGenome*. Distance to annotated sites is expressed in *sod* (sum of distances, ≥0). Note that in Ensembl annotation (GTF-files) the two exons are represented as two distinct features.

During annotation of query objects derived from genomic alignments, overlap with annotated genomic features is examined and (if existent) an optimal matching pair is selected. The annotation process for gap-sites is implemented in (CRAN) R-package *refGenome* (see Figure 2). First, intron-connected exon pairs need to be identified because exons are included in GTF file format (the format down-loadable from Ensembl or UCSC) as distinct entities only related by *transcript_id* and *exon_number*.

In a second step, a query list (containing gap-site data) and a reference list (containing positions of connected exons) are traversed simultaneously. During traverse, for each gap-site a region containing possible overlaps is searched for an optimal match (minimising *sod*).

Overlap is defined as the intersection of element-ranges in the query and the reference list. Element-ranges in the query list range from the first nucleotide with an alignment match (leftmost C in query3 in Table A1) to the last nucleotide with an alignment match (rightmost G in query1 in Table A1). An element-range in the reference list range from the start position of the left exon to the end position of the right exon. When no overlap is found (i.e., all intersections are empty), no annotation data is provided for a (query) gap-site (The R implementation in *refGenome* reports missing overlaps as “NA”-values (Not available).). The annotation procedure reports exact matches (sod = 0) as well as inexact matches (sod>0, Figure 2). Exact matches are gap-sites residing on annotated splice sites. Inexact matches may be due to biology, for example alternative splicing events (exon skipping, intron retention or alternative donor or acceptor sites) as well as splicing errors. However, inaccuracies may also be due to technical or bioinformatic issues like sequencing or alignment errors. 

### 1.6. Validation Strategy for Quality Scores

As no reference method is available for validating gap-sites (the biochemical RT-PCR method allows validation of small numbers of splicing events and is not feasible for genome wide analysis), a systemic approach using global statistical properties is utilised. The values which are statistically analysed reflect the complementarity to the 5${}^{\prime }$ end of U1 snRNA or known distribution of nucleotides around splice-sites. In detail, the used criteria are:

- Distribution of IDIN (GT or AG) and IDIN-pairs (GT-AT).
- Sequence logos from 5${}^{\prime }$ and 3${}^{\prime }$ exon-intron boundaries.
- The proportion of annotated sites.

## 2. Results

### 2.1. Global Statistics

#### 2.1.1. Alignments from TopHat Aligner

TopHat reported in total 3.0 × 10^9^ alignment gaps containing 1,000,380 gap-sites. Thus, gap-sites are in mean covered by 2999 alignments. Therefrom, 273,994 (27.4%) gap-sites are supported by only one alignment (nAligns = 1). TopHat reports 120,434 (12.0%) gap-sites present in all 54 samples and 243,596 (24.3%) annotated splice-sites (sod = 0).

The reported left IDIN (without strand information) are CT, GT, GC and AT and the right IDIN are AG, AC, GC and AT (both decreasingly ordered by abundance). In total, TopHat reports only gap-sites with 6 (from possible 256) different IDIN-pairs (GTAG, CTAC, GCAG, CTGC, ATAC, GTAT; decreasingly ordered by abundance).

**Figure 3:**
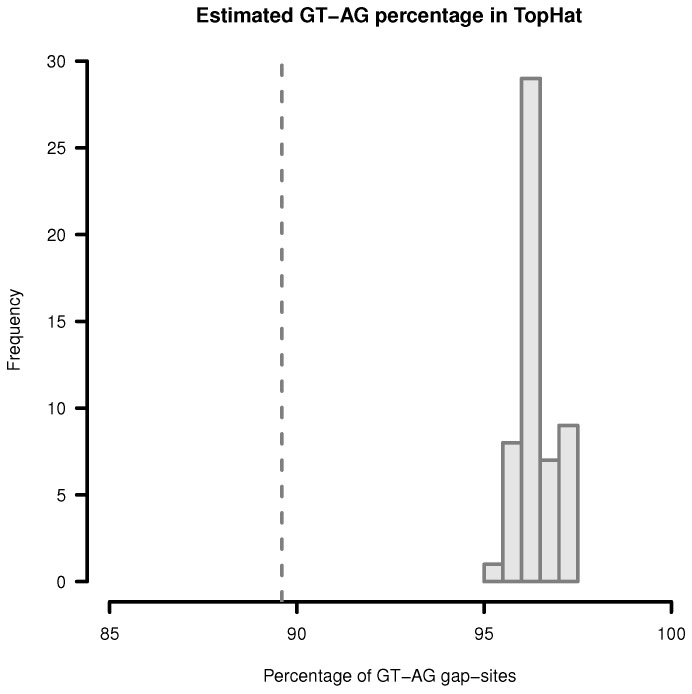
Estimation of *GT-AG*-site percentage in gap-sites reported by TopHat. Upper limit for percentage of *GT-AG* gap-sites (estimated by adding proportions of GT-AG and CT-AG) in TopHat alignments. Gap-sites were collected 54 times from single transcriptomes (vertical bars). Dashed vertical line (89.6%) indicates estimated percentage when gap-sites are collected from the complete batch of 54 transcriptomes.

In order to provide an upper limit for percentage of *GT-AG* gap-sites, the proportions of GT-AG and CT-AG sites are added. In single samples, the observed percentages vary in the range from 95.21% to 97.34% (Figure 3). The mean proportion is 96.4% (SD = 0.46%). When multiple samples are merged, the proportion of *GT-AG* sites will decline due to accumulation of noisy observations. The proportion in 54 merged transcriptomes is 89.6%. Thus, when multiple samples are merged, the proportion of GT-AG gap-sites can be expected to vary in the range between 89% to 98%.

#### 2.1.2. Alignments from STAR Aligner

STAR reported in total $2.4\times 10^{9}$ alignment gaps containing 6,487,577 gap-sites. Gap-sites from STAR aligner were supported in mean by 371 alignments. Therefrom 4,437,270 (68.4%) gap-sites are supported by only one alignment (nAligns = 1). STAR reports 129,758 (2.0%) gap-sites are present in all 54 samples and 256,044 (3.94%) annotated splice-sites. STAR reports all possible nucleotide combinations in left and right IDIN (STAR also allows gap-sites with IDIN’s containing N, for example AN and NT are present in left IDIN. From left IDIN, 31 were NN and from right IDIN, 74 were NN.).

**Figure 4:**
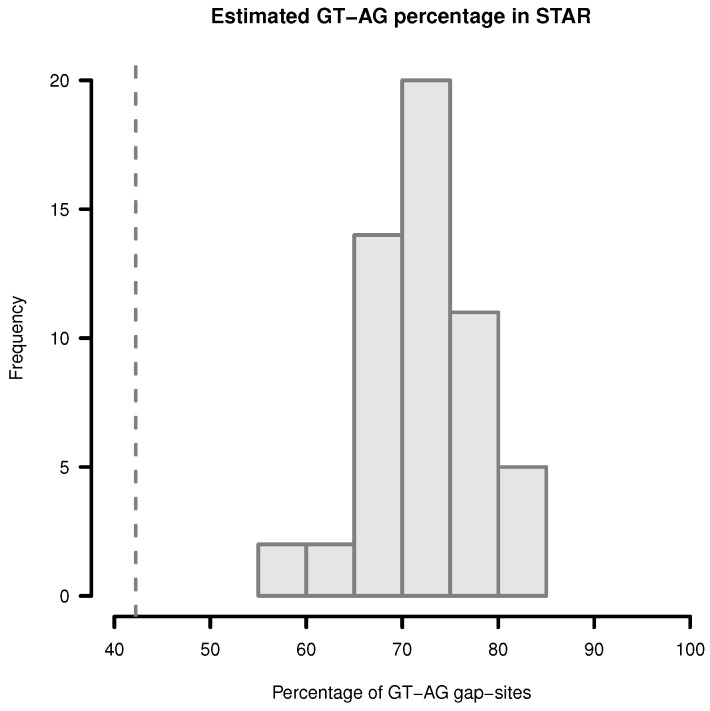
Estimation of *GT-AG*-site percentage in gap-sites reported by STAR. Upper limit for percentage of *GT-AG* gap-sites (added proportions of GT-AG and CT-AG) in STAR alignments. Gap-sites were collected 54 times from single transcriptomes (vertical bars). Dashed vertical line (42.2%) indicates estimated percentage when gap-sites are collected from the complete batch of 54 transcriptomes.

The proportion of *GT-AG* gap-sites in single samples (estimated by adding percentages of GT-AT-sites and CT-AG-sites) vary in the range from 57.72% to 82.35% (Figure 4). The mean proportion in single samples is 72.73% (SD = 5.43%). In merged data from 54 samples, 42.2% GT-AG (or CT-AG) sites are present. Thus, when multiple samples are merged, the proportion of *GT-AG* gap-sites can be expected to vary in the range between 42% and 83%.

#### 2.1.3. Comparison of Alignment Numbers

**Figure 5:**
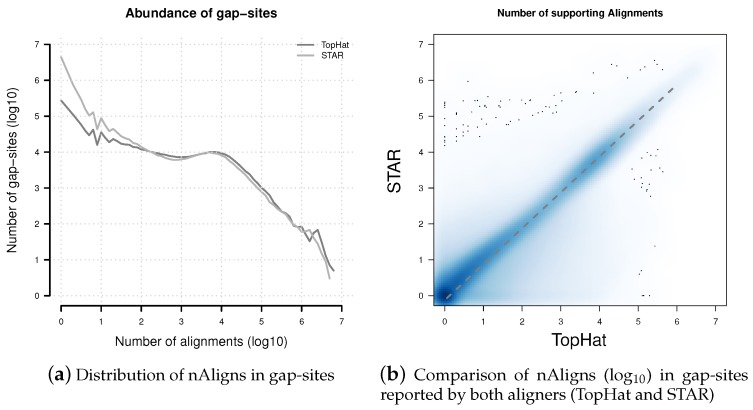
Distribution of nAligns values in gap-sites present in TopHat and STAR alignments. (**a**) Distribution of alignment coverage on gap-sites reported by TopHat and STAR; (**b**) Alignment coverage on gap-sites reported by both aligners (TopHat and STAR). The dashed line represents data from a linear regression model: nAligns_STAR_ = 0.756 × nAligns_TopHat_. Coordinates of both axes in both sub-figures are logarithmised (log_10_).

In general, the number of gap-sites decreases with higher alignment coverage (Figure 5a). For gap-sites with nAligns >100, the gap-site numbers essentially are distributed equally in TopHat and STAR alignments.

The STAR aligner reports substantially more gap-sites with low coverage (nAligns < 100): While TopHat reports 582,264 (58.2% of all alignments) with nAligns <10, STAR reports 5,939,689 (91.6% of all alignments) with nAligns <10, over 10 times more than TopHat (see Table 1).

There are 906,219 gap-sites which are reported by both aligners (TopHat and STAR). On these gap-sites, the nAligns numbers from STAR are approximately 76% of numbers from TopHat (Figure 5b). Thus, the in mean 10 times lower nAligns numbers in STAR alignments result only in a 24% decrease on single gap-sites and is mainly caused by a 10 times larger number of gap-sites with nAligns <100 (which are only partially also reported by TopHat).

### 2.2. Validation of gqs

#### 2.2.1. Distribution of *gqs*

**Figure 6:**
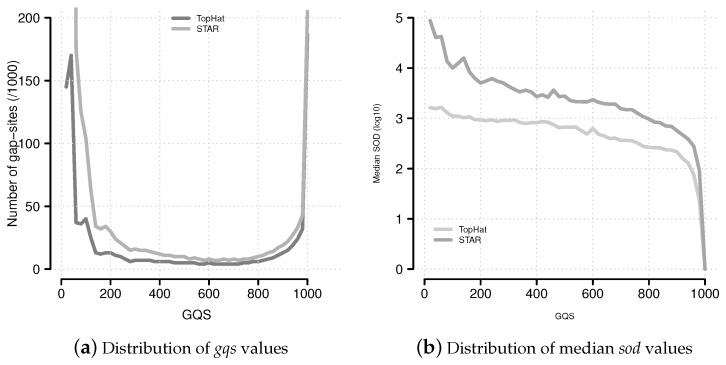
Distribution of *gqs* and *sod* values. (**a**) The distribution of *gqs* values follows a characteristic U-shaped pattern. This pattern is similar to the multiplicity of events (Supplemental Data Figure 9); (**b**) Median *sod* (distance of gap-site to annotated site) values for *gqs* categories.

The *gqs* values distribute in a characteristic U-like shape (Figure 6a). Alignments from STAR contain more gap-sites with *gqs* <200 than alignments from TopHat.

The median *gqs* value was 195 from TopHat alignments and 12 from STAR alignments. In order to achieve a median *sod* of 0 (equivalent to: >50% of gap-sites are annotated) via the *gqs*-based filter, a *gqs* of ≥990 is needed in TopHat-alignments and *gqs* = 1000 in STAR-alignments. Thus, a threshold of *gqs* = 1000 is used as threshold for *gqs*-validation.

#### 2.2.2. Number of *gqs*-Validated Gap-Sites

**Figure 7:**
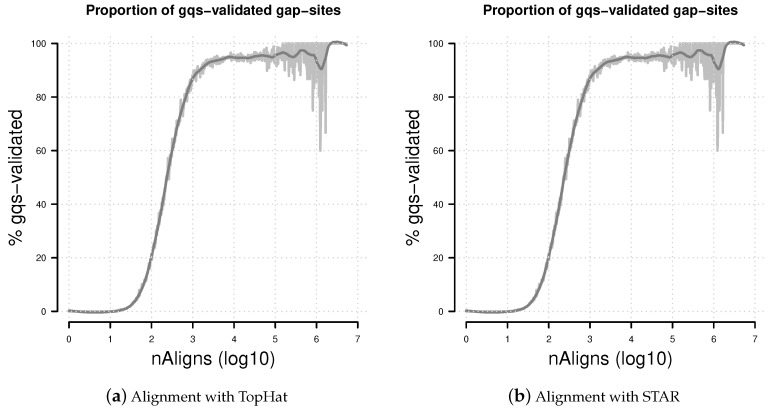
Percentage of *gqs*-validated gap-sites. Proportion of *gqs*-validated gap-sites for different alignment coverage’s. (**a**) Alignments from TopHat aligner; (**b**) Alignments from STAR aligner. The rising proportions of *gqs*-validated gap-sites reflects the fact that *gqs*-validation is more likely for higher alignment coverage.

In alignments from TopHat, 156,251 gap-sites were validated by *gqs* (18.5% of all reported gap-sites). In alignments from STAR, 166.294 gap-sites were validated by *gqs* (2.6% of all reported gap-sites). The proportion of *gqs*-validated gap-sites increases uniformly with alignment depth, producing a sigmoidal increasing line on $log_{10}$-transformed nAligns numbers (Figure 7). For validation of more than 50% gap-sites with a coverage of more than 468 alignments TopHat alignments and more than 240 alignments STAR alignments are required.

#### 2.2.3. Distribution of IDIN on *gqs*-Validated Gap-Sites

In *gqs*-validated gap-sites, the upper limit for the percentage of *GT-AG*-sites is 98.61% (49.54% + 49.07%) from TopHat alignments and 97.59% (49.13% + 48.46%) from STAR alignments (Table 2). The analogue calculation for splice-sites from the minor spliceosome (*AT-AC* sites) yields estimates of 0.25% for TopHat and 0.08% for STAR.

### 2.3. Validation of wgis

**Figure 8:**
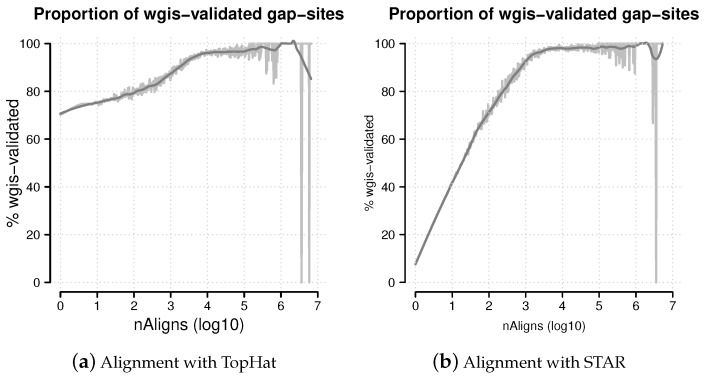
Percentage of *wgis*-validated gap-sites. Proportion of *wgis*-validated gap-sites for different alignment coverage’s (nAligns). Gap-sites were categorised by log_10_(nAligns) value (rounded by one digit). For each category, the proportion of *wgis* validated gap-sites were tabled. The raw proportions were smoothed using a *loess* model. (**a**) Alignments from TopHat aligner. The proportion of *wgis*-validated gap-sites is >70% throughout the whole range; (**b**) Alignments from STAR aligner. For nAligns >19 (log_10_(nAligns) >1.28), the majority of gap-sites (>50%) are validated by *wgis*.

**Figure 9:**
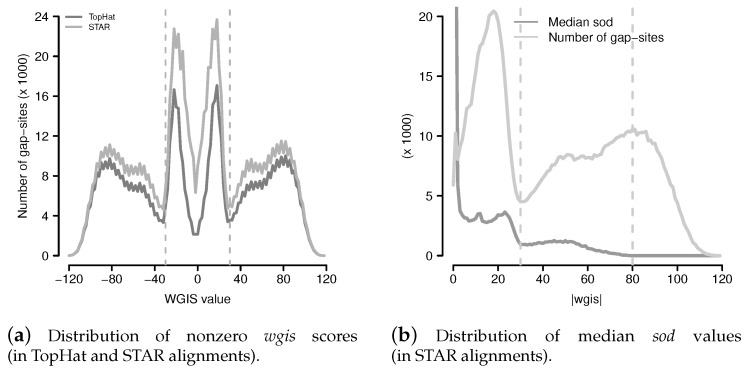
Distribution of *wgis* and median *sod* values. (**a**) Distribution of non zero *wgis* (*wgis* values were cut into ranges of width 2 for reduction of scattering); (**b**) Distribution of median *sod* and number of gap-sites from STAR with respect to absolute wgis values (|wgis|). Range limits are drawn at |wgis|=30 (local minimum of number of gap-sites) and at |wgis|=80 (where median *sod* drops to <10).

Observed values for *wgis* ranged from −119.2 to +118.7. In total, 1,083,629 gap-sites were validated by *wgis* in either TopHat or STAR alignments. In alignments from TopHat, 770,327 (77.0%) gap-sites were validated by *wgis*. In alignments reported by STAR, 1,065,596 (16.43%) gap-sites were validated by *wgis*. In general, the proportion of *wgis*-validated gap-sites increases with number of supporting alignments (nAligns, Figure 8). Using *wgis* provided strand information, 50.77% and 50.49% of *wgis* validated gap-sites were assigned to “+”-strand in TopHat and STAR alignments respectively. The *wgis*-distribution is almost identical in gap-sites assigned to “+”-strand and to “−”-strand (Figure 9).

#### 2.3.1. Definition of GQL Limits

Parallel evaluation of median *sod* values and number of gap-sites (Figure 9b) in STAR alignments shows that *wgis*-validated gap-sites appear to be a heterogeneous population separated by two natural limits:

- A limit at |wgis|=30, where number of gap-sites and *sod* have a local minimum.
- A second limit at |wgis|=80, where median *sod* drops to <10 in STAR alignments
  (Median *sod* = 0 for |wgis|>75 in TopHat alignments).

Together with the limit |wgis|>0 (separating *wgis*-validated from not validated gap-sites), four different groups, called *gql* (gap-site quality level) can be separated. Definition of *gql* and proportions of assigned gap-sites are shown in Table 3.

#### 2.3.2. Distribution of Intronic Dinucleotide Pairs

Distribution of intronic dinucleotide pairs in gap-sites with different *gql* levels are shown in Table 4. Intronic dinucleotide pairs associated with the minor spliceosome (*AT-AC*) constitute <1% of *wgis*-validated gap-sites.

### 2.4. Sequence Logos of Validated Gap-Sites

**Figure 10:**
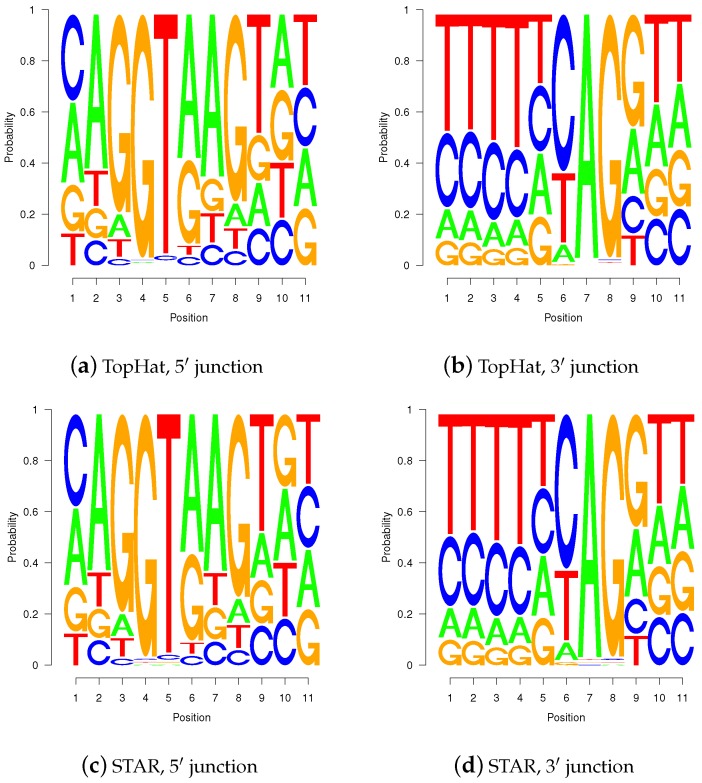
Sequence logos for *wgis* verified gap-sites. Sequence logos for *wgis* verified gap-sites (GQL > 0). Tabled nucleotides are corrected for strand orientation reported by *wgis*. (**a**,**c**) Splice-junction is located between position 3 and 4 (gap-site *lend* is located at position 3); (**b**,**d**) Splice-junction is located between position 8 and 9 (gap-site *rstart* is located at position 9). Sequence logos for *wgis* validated gap-sites show a high degree of similarity in alignments from TopHat and STAR.

Sequence logos of *wgis* validated gap-sites for 5${}^{\prime }$-junctions (Figure 10a,c) and for 3${}^{\prime }$-junctions (Figure 10b,d) show high similarity between TopHat and STAR alignments. The sequence logos show presence of the second GT at intronic position 5 and 6 in 5${}^{\prime }$ splice-junctions (positions 8 and 9 in Figure 10a,c) as well as pyrimidine rich 3${}^{\prime }$ terminal intronic regions (positions 1 to 6 in Figure 10b,d). The sequence logos are also highly similar to those calculated on annotated splice sites (shown in supplemental material).

### 2.5. Comparison of TopHat and STAR Alignments and gqs and wgis Validation

For comparison of validated gap-sites from TopHat and STAR aligner, tables with (*gqs* and *wgis*) validated gap-sites from TopHat and STAR were merged (using genomic coordinates as identity criterion).

#### 2.5.1. Global Statistics

In total, 6,581,738 gap-sites were identified by either TopHat or STAR in the 54 fibroblast samples. Therefrom, 5,581,358 (84.8%) are only present in STAR alignments, 906,219 (13.8%) are identified by both aligners and 94,161 (1.43%) are solely present in TopHat alignments (Table 5). From alignments reported exclusively by STAR, exclusively by TopHat or by both aligners, 97.6%, 82.9% and 65.0% of gap-sites are not validated by either *gqs* or *wgis*.

In result, STAR as well as TopHat do report gap-sites not seen by the other aligner. Some of these may even be validated by both scores.

#### 2.5.2. Relation between Score Value and Gap-Site Multiplicity

**Figure 11:**
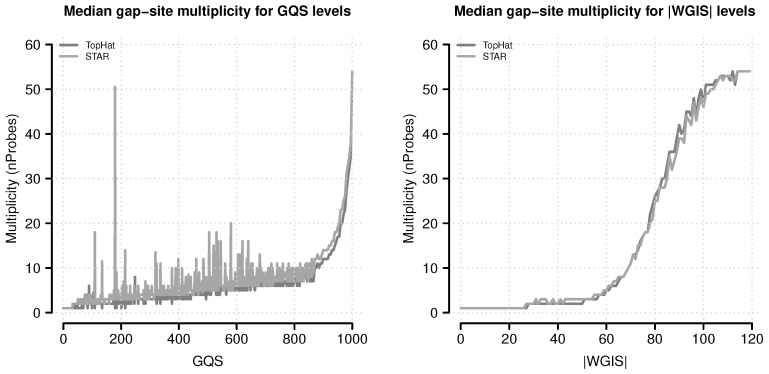
Median gap-site multiplicities for different score levels. Gap-site multiplicity (nProbes, the number of samples in which a gap-site is identified) are used as category (absolute values of *wgis* are rounded to integral numbers). For each score category, the median nProbes value is calculated and displayed in the figure. (**Left**) Gap-site multiplicities for different *gqs* values; (**Right**) Gap-site multiplicities for different |wgis| values.

The proportion of samples in which a gap-site is observed can be related to (absolute) score values (for *gqs* and |*wgis*|) which describe to which extent one criterion reflects the other one. Figure 11 shows that, in general, increasing score values (*gqs* and |*wgis*|) are associated with a higher proportion of samples in which a gap-site is identified.

There are gap-sites with low *gqs* which are present in a substantial fraction of samples. A high *gqs* is required (980 for TopHat and 975 for STAR alignments) in order to achieve presence in the majority (>50%) of samples.

Relations for |*wgis*| indicate a clearer relationship than observed for *gqs*. Gap-sites are present in the majority of samples (>50%) when their |*wgis*| value is >82. The median multiplicity becomes >1 when |*wgis*| is >26. Thus the gap-site multiplicities also support the *gql* classification criteria (In detail: The 50% limit is exceeded at |*wgis*| values of 81 in TopHat alignments and 82 in STAR alignments. The nProbes = 1 limit is exceeded at |*wgis*| values of 28 in TopHat alignments and 27 in STAR alignments.). Additionally, the straight and sigmoidal shape of the curve for |*wgis*| shows that *wgis* has a much better capability to predict gap-site multiplicity than *gqs*.

#### 2.5.3. Dependence of Gap-Site Validation on Gap-Site Coverage

**Figure 12:**
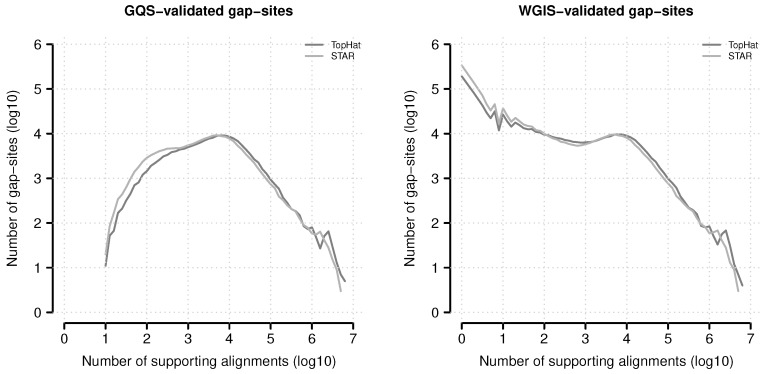
Absolute numbers of verified gap-sites for different alignment depth’s. Gap-sites were categorised according to number of supporting alignments (nAligns): The log_10_(nAligns) value rounded to one digit was used as category (x-axis). The lines display the log_10_ of tabled validated gap-sites (*gqs*-validated left, *wgis*-validated right).

The distribution of gap-site numbers in TopHat and STAR alignments verified by *gqs* or *wgis* for different levels of alignment coverage is shown in Figure 12. Globally, STAR reports 38.3% more *wgis* validated gap-sites than TopHat and 6.4% more *gqs*-validated gap-sites (More details are provided in supplemental material.). A (local) maximum of gap-site numbers is present at ≈$10^{4}$ alignments coverage, a value essentially determined by the total sequencing depth (on 54 fibroblast samples).

The majority of *wgis* validated and only a small minority of *gqs* validated gap-sites are supported by <100 alignments: For *gqs*-validation in 3.0% and 5.7% of gap-sites in TopHat and STAR alignments respectively. For *wgis*-validation in 74.1% and 71.4% of gap-sites in TopHat and STAR alignments respectively.

#### 2.5.4. Relation between *gqs* and *wgis* Validation

**Figure 13:**
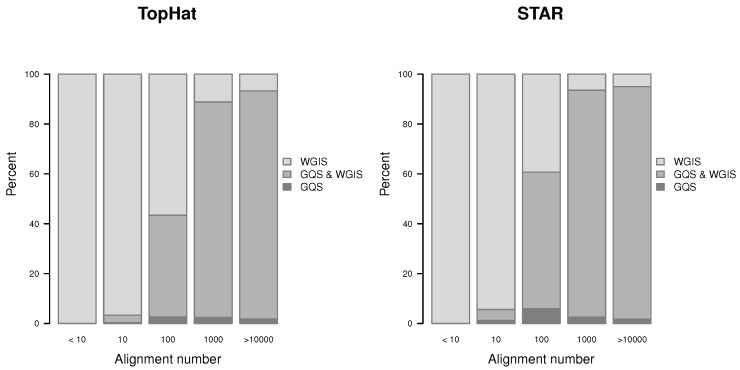
Proportion of verified gap-sites for different alignment depth’s.

Globally, the majority of verified gap-sites are verified by *wgis* alone (Table 5) but there may be differences in gap-sites with high alignment coverage. Therefore the verification with *gqs* and *wgis* is compared for different nAligns ranges (Figure 13). The percentage of gap-sites verified only by *gqs* is <3.2% in TopHat alignments and <6% in STAR alignments and thus very low.

The vast majority of gap-sites is verified by *wgis* alone or by both scores with the proportion of *gqs* validated sites increasing when alignment coverage is larger than 100. Proportions including gap-sites not validated by both scores (*gqs* and *wgis*) are shown in Supplemental material.

### 2.6. Unvalidated Gap-Sites

**Figure 14:**
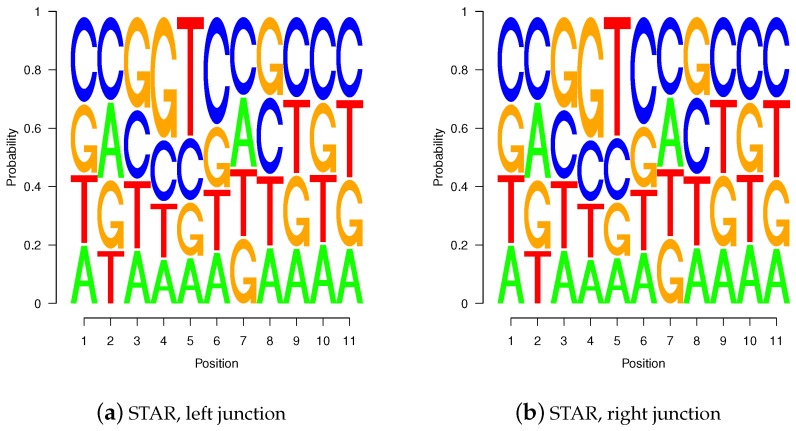
Sequence logos for gap-sites not validated by *wgis*. Sequence logos for (5,421,981) gap-sites not validated by *wgis* (GQL = 0) from STAR aligner. Tabled nucleotides are corrected for strand orientation reported by *wgis*. (**a**) Splice-junction is located between position 3 and 4 (gap-site *lend* is located at position 3); (**b**) Splice-junction is located between position 8 and 9 (gap-site *rstart* is located at position 9).

A gap-site is not *gqs* validated when *nlstart* < 8 or *qsm* < 200. A gap-site is not *wgis* validated when *qsm* ≤ 15 or when MaxEnt *score5* ≤ 1 or *score3* ≤ 1 in either strand direction. Low read coverage of gap-sites reduces likelihood of *gqs* validation while *wgis* validation still relies on MaxEnt scores as long as *qsm* > 15. While for *gqs*-validation, a coverage at least 8 alignments is required, *wgis*-validation can already be achieved with one single alignment. From *wgis*-unvalidated gap-sites 8431 (1.00%) in TopHat and 9803 (0.16%) in STAR alignments are found in all 54 samples. Also, TopHat and STAR report 8689 and 10,590 Ensembl annotated splice-sites respectively not validated by *wgis* as well as 7330 and 8186 splice-sites respectively not validated by *gqs*. Sequence logos for gap-sites reported by STAR aligner and not validated by *wgis* are shown in Figure 14. The sequence logos largely do not match splice-site consensus sequences demonstrating that validation is a critical prerequisite for further analysis.

#### 2.6.1. Maximal Alignment Coverage on Unvalidated Gap-Sites

In gap-sites not validated by *gqs*, the maximal alignment coverage is 2,994,298 in alignments from TopHat and 1,664,581 in alignments from STAR comprising 45.18% (TopHat) and 30.67% (STAR) of the maximal number of supporting alignments.

In gap-sites not validated by *wgis*, the maximal alignment coverage is 5,880,547 in alignments from TopHat and 3,545,564 in alignments from STAR comprising 88.73% (TopHat) and 65.34% of the maximal number of supporting alignments.

## 3. Discussion

### 3.1. Performance of Quality Scores

#### 3.1.1. *gqs*

Gap-sites collected from 54 fibroblast samples and validated by *gqs* contain a reasonable high proportion (≈98% in both aligners) of GT-AG sites. Using *gqs*, ≈156,000 gap-sites are validated in TopHat alignments and ≈ 166,000 in STAR alignments (6% more) in merged data from 54 samples. It may be advantageous that no additional information from genomic sequence is required for calculation of *gqs*. But for the validation of >50% of gap-sites, alignment coverage of more than ≈250–500 is required, a high number indicating limitations in sensitivity. This number is a static value independent of number of samples and depends solely on total alignment depth (on merged samples together). Also, *gqs* cannot provide strand information. Thus, validation of gap-sites with *gqs* shows, that suitable quality criteria can be derived from pure alignment data (BAM file content), but sensitivity is low and strand information must be provided from a second source which might be a challenging task.

#### 3.1.2. WGIS

The *wgis* uses the similarity of a gap-site with known splice-sites as quality criterion additionally to the alignment data. Using *wgis*, ≈770,000 gap-sites are validated in TopHat alignments and ≈1,066,000 in STAR alignments (38% more) in merged data from 54 samples. Thus, *wgis* validates 4.9 times (in TopHat alignments) and 6.4 times (in STAR alignments) as much as *gqs*. Approximately $\frac{3}{4}$ of gap-sites reported by TopHat are validated by *wgis*. Thus, the validation sensitivity is considerably higher in *wgis* than in *gqs*. Also, the strand information provided by *wgis* (without requirement of annotation) is a potentially valuable feature. Gap-sites validated by *wgis* can further be categorised into quality levels *gql*1 to *gql*3. Thus, usage of features in genomic sequence appears to be a valuable source of information for validation of gap-sites which may substantially increase sensitivity and also may provide strand information.

### 3.2. Comparison of TopHat and STAR Aligner

#### 3.2.1. Sensitivity and FDR

TopHat is known to have a low mapping yield while STAR has been shown to report many alignments with a high base-wise accuracy [3]. RGASP further found the rate of correctly mapped gapped-reads in the range of 96.3% to 98.4% but also notes at the same time, that many erroneously reported gap-sites are a major obstacle in STAR alignments [3].

For detection of occasionally observed splice sites and low abundant events, merging of data from multiple samples is necessary in order to increase sensitivity [19]. But usage of this approach increases sensitivity as well as FDR, possibly leading to *GT-AG* proportions of <50% in alignments from STAR. Also, the fact that 68.4% of unique gap-site positions (≈$4.4\times 10^{6}$ in our 54 samples) are only supported by one single alignment indicates a low specificity (high FDR) for STAR aligner.

An FDR in this magnitude diminishes reliability of downstream analysis. It is easily conceivable how high FDR for gap-sites deteriorate results of dependent functions like intron detection. Thus, we propose that for transcript reconstruction, a high FDR for gap-sites may be an impeding factor additionally to the difficulties imposed by the complexity of the human genome [2].

#### 3.2.2. Distribution of Gapped Alignments

In merged data from 54 RNA-seq samples, we found that although STAR aligner reports much more gap-sites than TopHat, the number of gapped alignments in STAR alignments actually is lower than in TopHat alignments. Thus, the higher mapping numbers reported by STAR (which partly are due to the ability to report truncated alignments [3]) seem to be not present in gapped alignments. Also, the higher number of gap-sites reported by STAR is mainly due to a different distribution of gapped alignments leading to a reduced alignment coverage on gap-sites (in mean 75.6% coverage in TopHat alignments). STAR reports 6.4% more *gqs*-validated gap-sites and 38.3% more *wgis*-validated gap-sites than TopHat. Thus the already reported higher sensitivity for STAR in general [3] is also present in (*gqs* or *wgis*) validated gap-sites.

### 3.3. Validation Strategies

As validation strategies for gap-sites may greatly enhance accuracy of the detection process, alternative strategies have already been proposed or implemented:

- Restrict alignment gaps to a small subset of possible intronic dinucleotides (as implemented in TopHat)
- Filter on alignment gaps supported by minimal 20 matching nucleotides on each side of the gap [3]
- Filter junctions on number of supporting alignments [3]
- Filter out alignments assessed as low confidence by regression model (basing on nucleotide distributions around splice junctions and intron size, as implemented in oLego) [20].

As the first filter bases on look-up of genomic sequence not present in BAM files and the second filter requires examination of CIGAR items adjacent to alignment gaps, both filters cannot be implemented with a simple algorithm. A filter simply basing on number of supporting alignments requires consideration of alignment depth (and a normalisation step). Also, in order to provide a consistent quality criterion, very high threshold values are required (at least 1.5 $\times 10^{6}$ alignments; see Section 2.6.1). Thus, using this criterion alone aggravates the low sensitivity of *gqs* for gap-sites with low coverage.

Based on considerations that the first three strategies “as is” are insufficient, we combined alignment based criteria into *gqs* and included sequenced based information using a complex criterion (MaxEnt) into *wgis*. The results indicate that criteria solely based on BAM-file content seem to lack sensitivity, but this handicap may be overcome by assessment of splice-site similarity. Criteria which are more sophisticated than simply filtering on IDIN-pairs even have the potential to detect rare splice-events possibly blurred by statistical noise.

#### Usage of MaxEnt Score

Filtering gap-sites based on MaxEnt scores may greatly enhance percentage of GT-AG-sites although this method might have limitations. First, evaluation of genomic sequence is not absolutely reliable for prediction of splice-site utilisation [21]. Also, MaxEnt in its current form assigns low scores to splice-sites recognised by minor spliceosome and ignores positions 10 and 11 of the 5${}^{\prime }$ splice-site. A correction cannot be introduced by shifting thresholds because this would lead to increased validation of gap-sites not similar to splice-sites recognised by the major spliceosome. Due to optimality properties of the MaxEnt estimation process, the only way for possible improvement strategies are:

- Calculation of new MaxEnt score tables basing on larger (or modified) “standard” samples.
- Creation of a second score solely basing on recognition by the minor spliceosome and using the maximum of both.

### 3.4. Limitations

Although it can be supposed, that reasonable proportions of nucleotide distributions (IDIN, IDIN-pairs and sequence logos) indicate that algorithms and decision rules act correctly in a majority of cases, these numbers should be interpreted with caution. As (potential) splice-sites are counted by occurrence and not by abundance, merging of data from multiple sources (for example by merging samples or by collecting data in an annotation data base) inevitably leads to rising numbers for rare events and thus may lead to overestimated proportions. The size of this effect is affected by the extensiveness of included information.

## 4. Materials and Methods

### 4.1. Fibroblast Samples

The transcriptome data analysed in this study originated from an investigation where effects of age, gender and UV exposition were studied in samples of dermal fibroblasts obtained from healthy human donors. A main result of the study is, that no consistent differential expression of genes is observable. The gene expression hence is assumed to be essentially homogeneous in these samples.

Details of sample preparation and the results differential expression analysis recently have been described elsewhere [12]. In short, the mRNAs of 54 samples were sequenced on an Illumina HiSeq 2000 sequencer yielding in total 8,785,501,333 reads. Subsequent alignments were calculated on unpreprocessed FASTQ files. Alignments were calculated using bowtie2 (2.2.5) [13], tophat (2.0.14) [14] and STAR (2.4.1d modified) [15]. Collection and processing of dermal samples from donors was approved by the Ethical Committee of the Medical Faculty of the University of Düsseldorf (# 3361) in 2011.

#### 4.1.1. Software

All described algorithms are implemented in R and are available from CRAN or Bioconductor. The software interface to samtools, the container and extraction algorithms for gap-sites are written in C/C++ and contained in CRAN package *rbamtools* [16]. Implementations for import of annotation data (GTF) and the annotation procedure for gap-sites are written in C/C++ and contained in CRAN package *refGenome*. Implementations for calculation of MaxEnt scores were translated into C and are publicly available in Bioconductor package *spliceSites* (version ≥ 1.23.5). Algorithms for extraction of DNA sequence for exon/intron boundaries use Bioconductor framework (Biostrings). The source code for R (for analysis and package development) as well as the latex source code for this document was developed using RStudio [17].

#### 4.1.2. Statistical Evaluation

For *wgis*-validated gap-sites, IDIN are reported after correction for strand orientation. Strand assignments are calculated as described in Section 1.3.2 (the *wgis*-reported strand). In sequence logos, letter height corresponds to nucleotide frequencies at given positions. Nucleotides are decreasingly ordered according to their frequency from top to bottom. All shown sequence logo’s are calculated after correction for strand orientation.

## 5. Conclusions

The aligners TopHat and STAR report a high rate of unvalidated gap-sites emphasising the necessity for further validation. Validation of gap-sites without using genomic sequence data (*gqs*) requires high alignment coverages which can greatly be reduced when the similarity to known splice-sites is evaluated.

## Figures and Tables

**Table 1:** Global statistics for TopHat and STAR aligner.

		Gap-Sites			
Aligner	Alignment Gaps	Number	Coverage	Single Align	All Samples
TopHat	2,999,472,708	1,000,380	2999	273,994	120,434
STAR	2,410,424,541	6,487,577	371	4,437,270	129,758

Global statistics for TopHat and STAR aligner: Alignment gaps (Total number in alignments), Gap-sites (Total number of unique gap-sites identified in 54 samples), Coverage (Alignment gaps/gap-sites = mean alignment coverage), Single align (Total number of gap-sites covered by one single alignment), All samples (Number of gap-sites present in all 54 samples).

**Table 2:** Percentage of intronic dinucleotides on *gqs*-validated gap-sites.

Left IDIN				Right IDIN				Combined		
IDIN	TopHat	STAR		IDIN	TopHat	STAR		IDIN	TopHat	STAR
GT	49.65	49.26		AG	50.10	49.68		GT-AG	49.54	49.13
CT	49.65	49.09		AC	49.20	48.60		CT-AC	49.07	48.46
GC	0.56	0.61		GC	0.59	0.60		CT-GC	0.59	0.54
TG		0.06		GG		0.16		GC-AG	0.56	0.48
CA		0.09		CC		0.14		AT-AC	0.14	0.04
AT	0.14	0.08		AT	0.11	0.11		GT-AT	0.11	0.04

Percentage of intronic dinucleotides (IDIN) and intronic dinucleotide pairs (IDIN-pairs) on *gqs*-validated gap-sites. The shown IDIN and IDIN-pairs are not corrected for strand orientation. IDIN are counted separately on data from each aligner (TopHat and STAR). Nucleotides represent untransformed genomic sequence (no correction for strand orientation). The numbers in each column sum up to 100 (TopHat) or ≈100 (STAR).

**Table 3:** *gql*: Definition and distribution.

						TopHat			STAR		
*gql*	*wgis*					N_total_	P_total_ (%)	P_val_ (%)	N_total_	P_total_ (%)	P_val_ (%)
0			|wgis|	=	0	230,053	23.0		5,421,981	83.6	
1	0	<	|wgis|	≤	30	256,950	25.7	33.4	446,316	6.9	41.9
2	30	<	|wgis|	≤	80	331.955	33.2	43.1	416,316	6.4	39.1
3	80	<	|wgis|			181,422	18.1	23.6	202,964	3.1	19.1

Definition of *gql* levels and assignment of gap-sites (collected from 54 fibroblast samples using STAR aligner). N_total_: Absolute number of gap-sites. P_total_: Proportion of all gap sites in percent. P_val_: Proportion of validated gap-sites in percent.

**Table 4:** Strand corrected intronic dinucleotide pairs of *wgis*-validated gap-sites.

IDIN-Pair	TopHat			STAR			Literature
	*gql*1	*gql*2	*gql*3	*gql*1	*gql*2	*gql*3	
*GT-AG*	95.70	98.71	100.00	94.76	98.66	100.00	99.24 %
*GC-AG*	3.83	1.24		3.38	1.15		0.7 %
*GT-AT*	0.23	0.03		0.16	0.02		
*AT-AC*	0.04			0.02			0.05%
*CT-AC*	0.20	0.02		0.54	0.02		

Percentage of intronic dinucleotide pairs in alignments from TopHat and STAR (IDIN-pairs not reported by TopHat not shown). Strand assignment solely bases on *wgis* (on MaxEnt *score3*). Percentage values from literature are taken from [18]. Empty spaces indicate proportion <0.005%. Obviously *GT-AT* sites and *CT-AC* sites are assigned to the wrong direction.

**Table 5:** Validation numbers of gap-sites.

Aligner	Not Validated	GQS	WGIS	GQS & WGIS	Sum
STAR	5,449,996	5548	125,217	597	5,581,358
STAR & TopHat	587,008	22,798	161,127	135,286	906,219
TopHat	78,044	680	15,164	273	94,161
Sum	6,115,048	29,026	301,508	136,156	6,581,738

STAR: Gap-sites identified only by STAR, TopHat: Gap-sites identified only by TopHat, STAR & TopHat: Gap-sites identified by both aligners. GQS: Gap-sites validated only by *gqs*, WGIS: Gap-sites validated only by *wgis*, GQS & WGIS: Gap-sites validated by *gqs* and *wgis*. Number of gap-sites for different validation types and presence in alignments. For gap-sites reported by both aligners, validation numbers were taken from STAR alignments. For these gap-sites, nAligns values are in mean ≈25% larger in TopHat alignments than in STAR alignments. (Larger *nlstart* and *qsm* values result in more validated gap-sites. Due to identical genomic coordinates, the MaxEnt scores are equal in both alignments).

**Figure A1:**
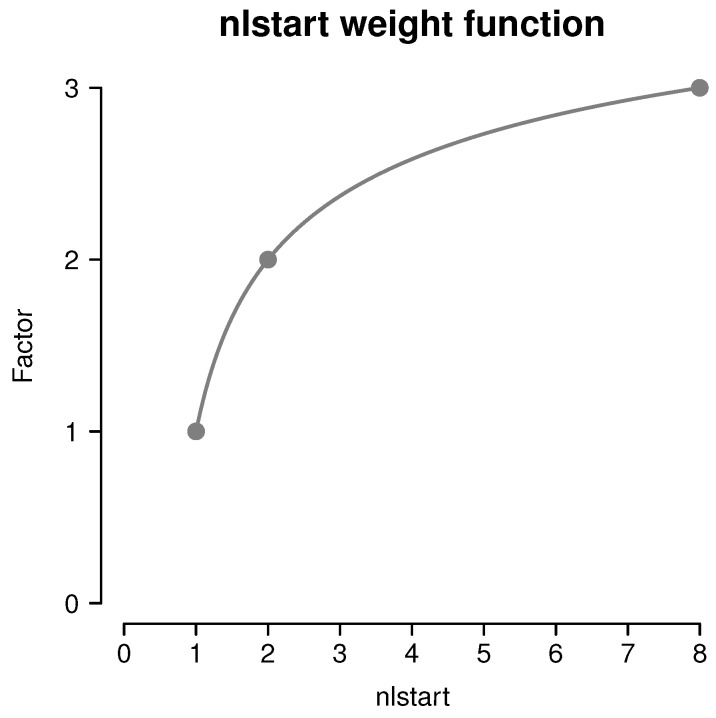
Weight function for *nlstart*. Occurrent values for *nlstart* are values in {1,…,8}. The weighted value for *nlstart* is defined as $\log_{2}\left(\log_{2}\left(nlstart\right)+1\right)+1)$. Points are added at *nlstart* values of 1, 2 and 8 (indicating weighted values of 1, 2 and 3 respectively).

**Figure A2:**
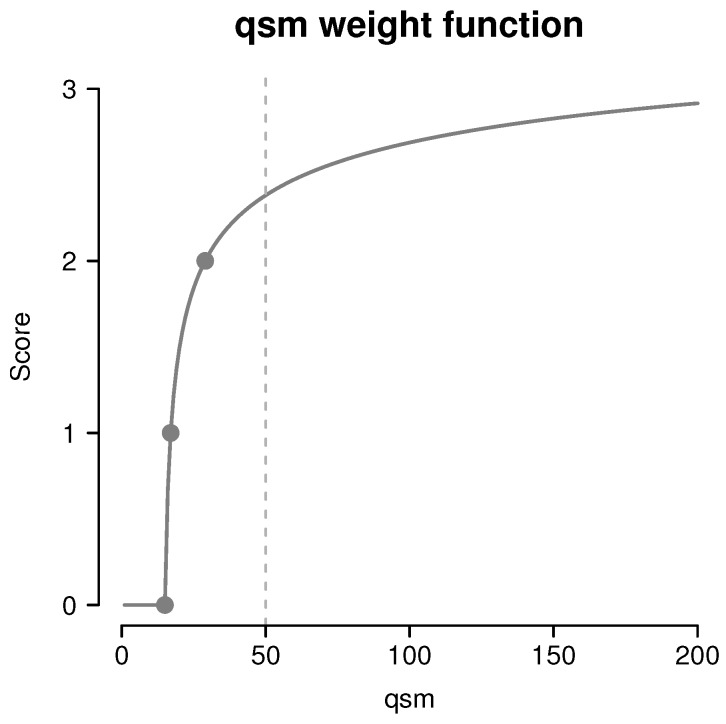
Weight function for *qsm*. Collected *qsm* values are integral values between 1 and (2× read-length). The weighted value for *qsm* is defined as $\log_{2}\left(\log_{2}\left(\max\left(qsm-13,2\right)\right)\right)$. Points are added at *qsm* values of 15, 17, and 29 (indicating weighted values of 0, 1 and 2 respectively).

## Supplementary Materials

Supplementary materials can be found at www.mdpi.com/1422-0067/18/6/1110/s1.

## Abbreviations

FDR	False discovery rate
nAligns	Number of supporting alignments (for a gap-site)
*gqs*	Gap quality score
*wgis*	Weighted gap information score
IDIN	Intronic dinucleotides
*mcl*	Minimum CIGAR length
*qsm*	Quartet sum of MCL
*lend*	Left end
$\log_{2}$	Logarithm to the base 2
$\log_{10}$	Logarithm to the base 10
*rstart*	Right start
*nlstart*	Number of left start (positions)
RGASP	RNA-seq Genome Annotation Assessment Project
SD	Standard deviation

## Appendix A. Information Collected for Gap-Sites

### Appendix A.1. BAM File Format

Gap-sites are identified using alignment data provided in BAM (Binary Alignment/Map) format (see also https://samtools.github.io/hts-specs/SAMv1.pdf). In a BAM file, each alignment is represented by a set of data items, mainly the reference sequence (chromosome number), the position and CIGAR items.

#### Appendix A.1.1. Gap-Site Positions Extracted from BAM File Format

Multiple gapped alignments define a single gap-site when they contain identical located alignment gaps. The genomic position of the rightmost matching nucleotide on the left side of the alignment gap is named *lend* (left end). The position of the leftmost matching position on the right side of the alignment gap is named *rstart* (right start). In the following example from a single genomic alignment

01234567890123456
CCCTACGTCCCAGTCAC (reference)
TAC TCAC (query)

the significant genomic positions are lend = 5 and rstart = 13. *lend* and *rstart* are the defining positions of a gap-site. The *gap-length* is defined as the number of nucleotides which are not covered by alignment matches. In our example, the gap-length is 7 (=rstart−lend−1).

#### Appendix A.1.2. CIGAR Items in BAM File Format and Derived Values

Details of alignments, for example match length or gap-size, are provided in the CIGAR string for each alignment. A CIGAR string consists a sequence of CIGAR items. Each CIGAR item consists of number (the length or number of affected nucleotides) and a single character (the type of “CIGAR operation”). The CIGAR item 50 M represents a subsequent match of 50 nucleotides (between query and reference sequence). The CIGAR item 1000 N indicates an alignment gap of length 1000. An alignment gap is represented by triple sequence of CIGAR items xMyNzM where y is the gap-length. Examples are shown in Table A1. Multiple alignment gaps sharing the same *lend* and *rstart* position (on the same reference sequence) constitute a gap-site. The gap-site sharing alignments are called “supporting alignments”. The values *nlstart* and *qsm*, used in *gqs* and *wgis*, are directly calculated from CIGAR strings in all supporting alignments.

**Table A1:** Gapped alignments and gap-sites.

Name	Match	Gap	Match	Position	CIGAR	*mcl*
query1	AG		CCTTGATG	3	2M6N8M	2
query2	CAG		CCTTGAT	2	3M6N7M	3
query3	CCAG		CCT	1	4M6N3M	3
reference	CCCAG	GTCCAG	CCTTGATGTCC			

In the gap-site defining alignments, three different match lengths (=CIGAR length for M-item) on the left side of the alignment gap are present (values 2, 3 and 4). Therefore, the *nlstart* value for the shown gap-site is 3. The *mcl* values for the three alignments are 2, 3, 3 thus resulting in a *qsm* of 8 (the missing *mcl* value is set to 0). The *gqs* resulting from the shown alignments is 15.

#### Appendix A.1.3. The *mcl* Value of a Gap-Site

The minimum CIGAR length (*mcl*) value is defined as the minimum of adjacent match lengths (M item in CIGAR segment) on each side of the alignment gap (N item in CIGAR segment). Thus, an *mcl* value of 10 for an alignment gap defined by CIGAR items *x*M*y*N*z*M ensures that x≥10 and y≥10 ensuring, that the gap at least is supported by 20 subsequent nucleotide matches in query (read) sequence.

#### Appendix A.1.4. The *qsm* Value of a Gap-Site

From alignments defining a gap-site, the quartet sum of mcl (*qsm*) is calculated as the sum of the four largest *mcl* (see Table A1) values from alignments defining the gap-site. Assuming, a gap-site is defined by n alignments with *mcl* values ${\left(mcl_{i}\right)}_{i=1\ldots n}$ and that the *mcl* values are ordered decreasingly (i.e., $mcl_{i}\geq mcl_{i+1}$) the definition can be stated as

(A1) $$qsm:=\sum_{i=1}^{4}mcl_{i}.$$

For read lengths of 101, the maximal value of *qsm* from one alignment can be 50 and the maximal *qsm* value of 200 can be achieved with at least four alignments. The *qsm* provides a measure for the amount of coverage based information supporting the presence of a splice site. As maximal four alignments account for *qsm*, information from long continuous alignments is emphasised.

#### Appendix A.1.5. The *nlstart* Value of a Gap-Site

The *nlstart* value of a gap-site is the number of different match lengths in the left adjacent matching segment of alignments defining a gap-site. When a gap-site is defined by n alignments ${\left(A_{i}\right)}_{i=1,\ldots ,n}$ containing CIGAR items $x_{i}My_{i}Nz_{i}M$, the *nlstart* value is defined as the number of different $x_{i}$ values. In Table A1, the $x_{i}$ values are {2,3,4}, so *nlstart* = 3.

Regarding the $x_{i}$ values as realisations of a random variable where all observed values occur with equal probability, and using the definition of Shannon entropy, it is known that log2(n) provides a measure of information content of the values $x_{i}$ (i.e., the number of bits necessary for encoding the $x_{i}$ values).

In a discrete probability space A=(A,p) where *A* is a finite set ($A=\{a_{1},\ldots ,a_{n}\}$) and *p* is a probability measure ($p\left(a_{i}\right)=:p_{i}$), the *Shannon entropy* of *A* is defined as

(A2) $$H\left(A\right):=-\sum_{i=1}^{n}p_{i}\log_{2}\left(p_{i}\right).$$

In case of equal probabilities ($p_{1}=\ldots =p_{n}$) the entropy reduces to $H\left(A\right)=\log_{2}\left(n\right)$.

## Appendix B. Weight Functions for *qsm* and *nlstart*

The *wgis* applies weight functions to *nlstart*, *qsm* and MaxEnt values. From the monotonicity criterion follows, that only monotone increasing weight functions may be used. In order to emphasise information contained in few high quality alignments (which increases sensitivity), the weight functions must be concave (having monotonic decreasing slope) advocating the logarithm function.

The logarithm for base *a* ($log_{a}$) solely adds a constant factor (log(a)) to the natural logarithm (See Supplemental Material). As the shape of the function is unchanged, the decisive properties of a score are equal for all base values. Thus, the logarithm base can be chosen arbitrarily (>0). For ease of calculation, $\log_{2}$ is utilised for all weight functions.

### Appendix B.1. Weighting of nlstart value

The logarithmic weight for *nlstart* values emphasises gap-sites constellations with more then one alignment start position. *nlstart* values of 2 and 8 are translated into *nlstart*-factor values of 2 and 8 respectively.

### Appendix B.2. Weighting of qsm Value

A major obstacle for alignment based validation of splicing events is a too small number of aligned nucleotides (on either side of the gap-site). Therefore, a lower limit for the number of aligned nucleotides on either adjacent region is introduced, implemented as lower limit for *qsm*. The weight function for *qsm* is constructed so that *qsm* values of 17 and 29 result in a *qsm*-factor of 1 and 2 respectively. The maximal possible *qsm*-factor (for reads of length of 101) is 2.91. The *qsm* weight function describing the relation between *qsm* and *qsm*-factor is shown in Figure A2.
