# Supplementary material for: Validation of Splicing Events in Transcriptome Sequencing Data
Source: Int J Mol Sci. 2017 May 23;18(6):1110. doi: 10.3390/ijms18061110 (PMC5485934; doi:10.3390/ijms18061110)
Supplement: Supplementary file 1 [file ijms-18-01110-s001.pdf]

Supplemental material

Validation of splicing events in transcriptome sequencing data

Wolfgang Kaisers[1], Johannes Ptok [3], Holger Schwender[2], Heiner Schaal[3]
[1] Department for Anaesthesiology; kaisers@med.uni-duesseldorf.de
[2] Mathematical Institute; schwender@math.uni-duesseldorf.de
[3] Institute for virology; schaal@uni-duesseldorf.de
[1]kaisers@med.uni-duesseldorf.de

Contents

1 Alignment of RNA-seq data **2**
1.1 Nomenclature . . . 2
1.1.1 Coordinates on genomic sequences . . . 2
1.2 Genomic alignments . . . 2
1.2.1 Gapped alignments . . . 2
1.2.2 Data volumes . . . 2

2 Threshold criteria for gap-sites **3**
2.1 Empirical base for selection of thresholds . . . 3
2.2 Empirical base for *qsm* threshold . . . 3
2.3 Consideration on intron size based filters . . . 3
2.4 Median SOD . . . 4

3 MaxEnt scores **6**
3.1 Distribution of MaxENT scores . . . 6
3.2 Usage of both scores in *wgis* . . . 6
3.3 Calculation of strand orientation from MaxEnt scores . . . 6
3.3.1 Limitations of calculation model . . . 6
3.3.2 Selection of score for estimation of strand orientation . . . 6
3.4 Empirical base for MaxENT thresholds . . . 6
3.5 Distribution of MaxENT scores on splice sites from minor spliceosome . . . 6

4 Annotated splice sites **8**
4.1 MaxEnt scores for annotated splice-sites . . . 8

5 Properties of *gqs* and *wgis* **10**
5.1 Global sample properties . . . 10
5.2 Comparison of *gqs* and *wgis* validation . . . 10
5.2.1 *gqs* and *wgis* validation of high abundant gap-sites . . . 10
5.2.2 *gqs* and *wgis* validation of low abundant gap-sites . . . 10
5.2.3 Alternative validation limits for *gqs* . . . 10
5.2.4 Proportion of samples in which gap-sites are identified . . . 10
5.2.5 Gap-sites present in all samples . . . 10
5.3 Analysis for GQL levels . . . 14
5.3.1 Sequence logos . . . 14

6 Total number of splicing events in human tissues **15**

7 Gap-sites with high alignment coverage **16**

8 Miscellaneous remarks **16**
8.1 Logarithm for base *a* . . . 16

1 Alignment of RNA-seq data

1.1 Nomenclature

1.1.1 Coordinates on genomic sequences

Reference genomes are organised in *reference sequences* (chromosomes) consisting of a successive ordering of nucleotides. Nucleotide sequences in a reference are read from left to right, (corresponding to the + strand and 5' to 3' reading direction). Genomic positions of nucleotides are given in in ascending order from left to right beginning with 0 (i.e. 0-based coordinates).
A genomic *range* is a set of consecutive nucleotides in a reference sequence. The location of a range is given by the a pair of coordinates: The leftmost and rightmost nucleotide belonging to the range. The coordinates are denoted *start* and *end* respectively.

1.2 Genomic alignments

The result of alignment procedures are coordinates of genomic nucleotides matched to sequencing read nucleotides usually given by a set of genomic ranges. Sequence alignments usually are reported in BAM file format [4]. CIGAR items describe alignment details in BAM files [1]. For example the CIGAR item 100M describes a subsequent match of all nucleotides of a read of length 100. For each alignment, chromosomal start position (0-based) and CIGAR items are reported.

1.2.1 Gapped alignments

Gapped alignments cover splice sites. A splice site consists of genomic locations of two exons and an enclosed intron.

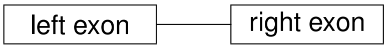

Therefore, in a gapped alignment, two matching regions are separated by an alignment gap (the intron).
In a BAM file, an alignment gap could be reported as 20M200N80N meaning that, from the start position, there are 20 matching nucleotides followed by a gap of 200 nucleotides and a second matching region of 80 nucleotides size. Altogether the length of the entire read sums up to 100.
Because sequencing reads usually do not cover entire en-framing exons of spanned introns, the positions of the leftmost and rightmost matching nucleotides are random and not significant. On the other hand, borders of enclosed alignment gaps indicate exon-intron boundaries and thus are significant. Gap-sites represent putative splice-sites but not all gap-sites represent true splice-sites, so candidates for real splicing events must be filtered out.
Per definition, each gap-site is covered by at least one alignment. The number of alignments sharing a gap-site is called given by a value called *nAligns*.
The number of samples in which a gap-site has been identified is given by the *nProbes* value.

1.2.2 Data volumes

Identification and validation of splicing events in RNA-seq data imposes several challenges due to large data volumes associated with RNA-seq analysis. For detection of 80 % of alternative splicing events alignment depths of 50 to 100 are required [4] resulting in read numbers the range of $100-150\times 10^6$. Therefrom the volume of compressed FASTQ data and of compressed alignment data (BAM file format) is in the range of 10 - 15 Gigabytes per sample. Thus alignments and samples must be processed sequentially in order to avoid extraordinary large demands for RAM (computer working memory). As BAM files can only be sorted according according to alignment position (and alignment name) and not by gap-site position, data insertion into the interior of a container may be necessary. Also the total number of gap-sites (is not known in advance and possibly varies between a few thousand and millions. Therefore gap-site data must be kept in a linked-list (and not a static array). Fortunately, the total number of gap-sites is in a range which can easily be stored in the RAM of a standard computer.

[1] See SAM/BAM format specification for details: https://samtools.github.io/hts-specs/SAMv1.pdf

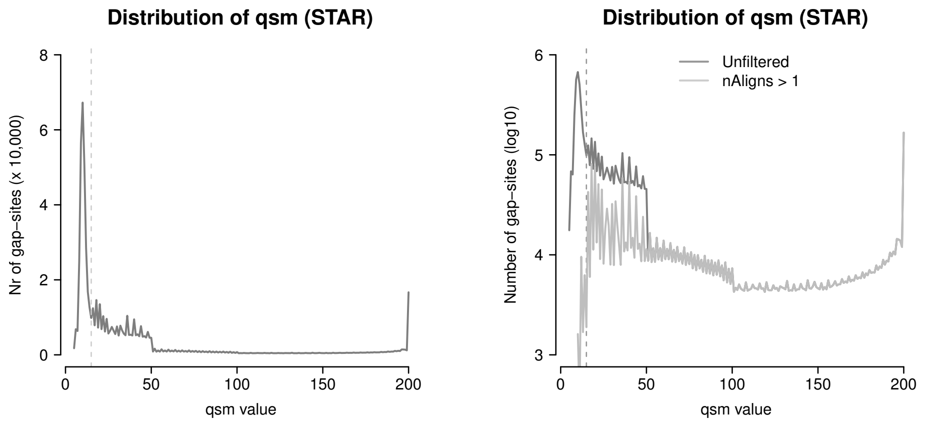

Figure 1: Distribution of *qsm* (alignments by STAR)

Distribution of *qsm* values from alignment with STAR. *qsm* threshold of 15 is marked by dashed line. **Left:** Number of gap-sites (in 10,000). **Right:** Total and number of gap-sites and number of gap-sites covered by $>$ alignment transformed by log10. The majority of gap-sites excluded by criterion *qsm*$>$ 15 is covered by only one alignment.

2 Threshold criteria for gap-sites

2.1 Empirical base for selection of thresholds

Thresholds for *qsm* and MaxENT scores had been chosen based on distribution of values on all (unfiltered) gap-sites.

2.2 Empirical base for *qsm* threshold

A threshold of 15 for *qsm* values had been set to the upper limit of a peak consisting of low confidence gap-sites. Application of this limit results in exclusion of 2,800,929, 43.1 % of all (6,487,577) gap-sites. The vast majority of excluded gap-sites (99.2 %) is supported by only a single alignment.

2.3 Consideration on intron size based filters

Implausible size of gap-length may be a reason for exclusion of a gap-site from further consideration. Here, the size of annotated introns in Ensembl annotation could provide en empirical base for a sensible limit. As this criterion is not related to any information on number of alignments or number of aligned nucleotides or sequence complementarity, limits used for exclusion should be selected conservatively in order to avoid unacceptable false negative results. The distribution of Ensembl intron sizes in shown in Figure 2. In all examined Ensembl versions, the 95 % quantile of intron length was <37,000 and the 99 % quantile was <120,000. From the total number of gap sites, 39.8 % of sites were >120,000. When using the wgis filter (WGIS $\neq$ 0), only 10.86 % of the remaining gap-sites exceed the value of 120,000 in our fibroblast data. Therefore, only a minor portion of sites would additionally be filtered out using a gap-length filter of 120,000. Also, a gap-length based filter could easily be applied afterwards. Therefore, no intron size based information has been included into *gqs* and *wgis* scores.

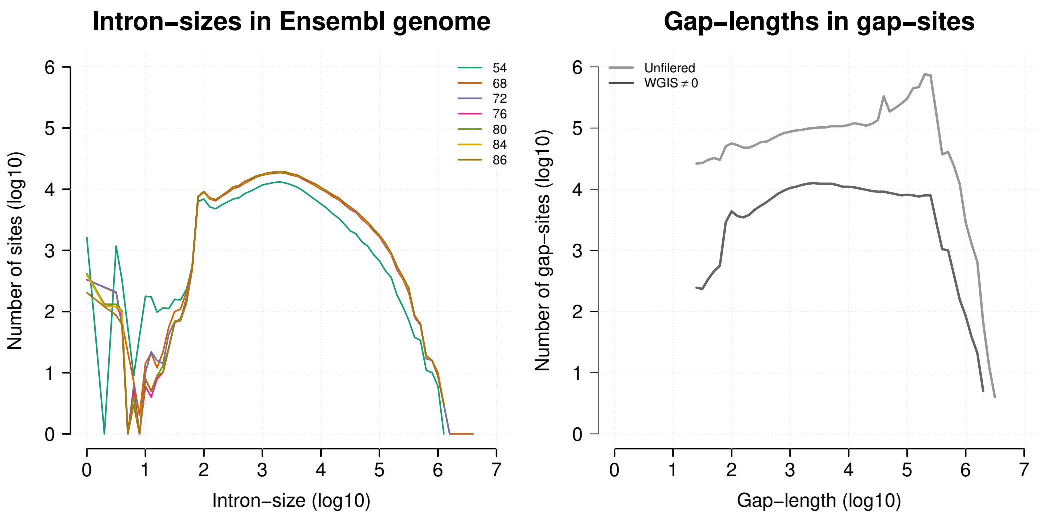

Figure 2: Distribution of intron size

Left: Distribution of intron size in Ensembl annotation. Intron size was calculated from downloaded GTF files using *refGenome* package. **Right:** Distribution of gap-length in gap-sites (from alignment with STAR aligner).

2.4 Median SOD

The definition of *gql* is derived from number of gap-sites and *sod* values from alignments with STAR (see section 3.3 Validation of *wgis* and Figure 8 of main document). The data shown in Figure 10 B in the main document was also extracted from TopHat alignments (shown in Figure 3).
Included are the *gql*-limits shown as vertical dashed lines (at $|wgis| = 30$ and $|wgis| = 80$).
In alignments from TopHat, median *sod* values are zero for all gap-sites with $|wgis| > 75$.

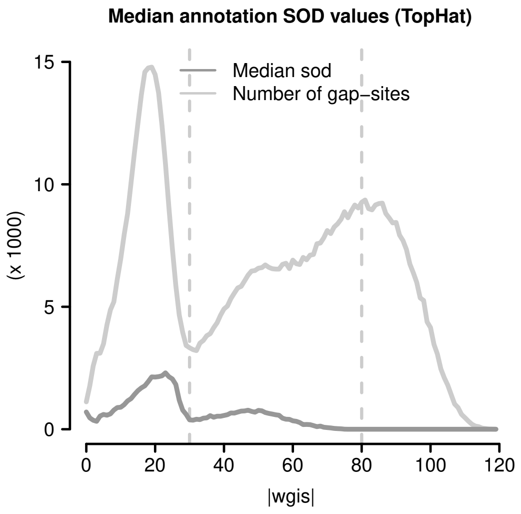

Figure 3: Median sod values (TopHat)

Median sod values from TopHat alignments. The corresponding figure for STAR alignments is shown in the main document.

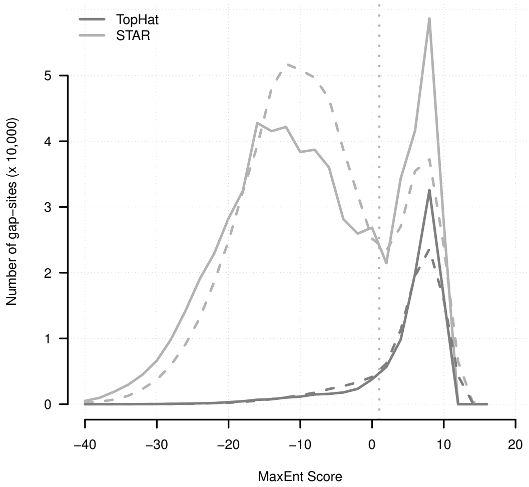

A: MaxEnt on all gap-sites

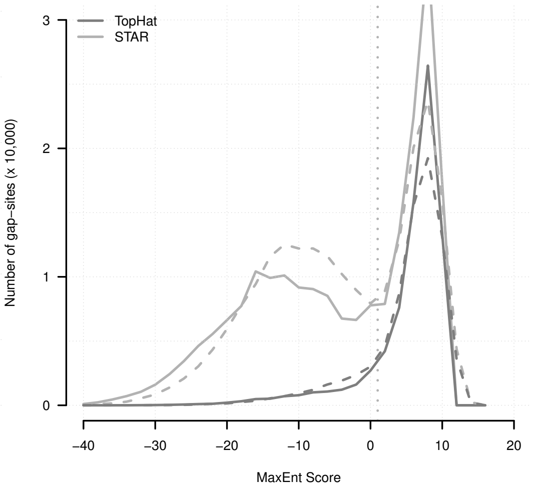

B: MaxEnt for Max nAligns >1

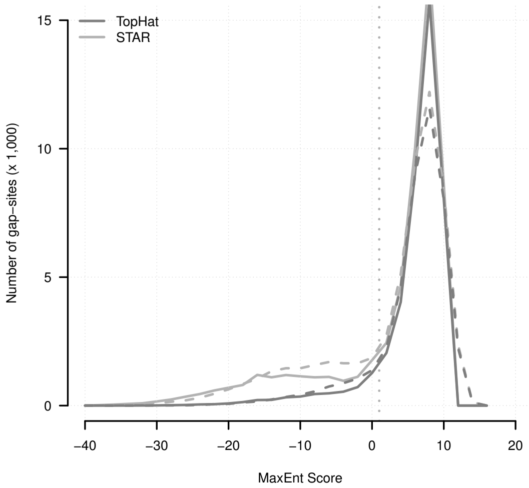

C: MaxEnt for Max nAligns >10

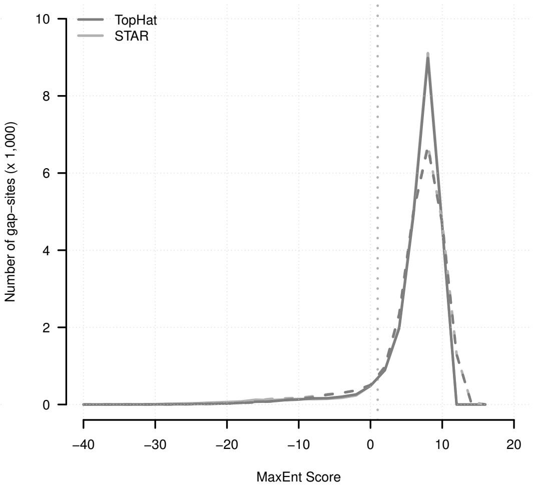

D: MaxEnt for Max nAligns >100

Figure 4: Distribution of MaxENT scores

Solid line: *score5*, Dashed line: *score3*. The dotted vertical line marks the position of the threshold (=1). For each gap-site the maximum of nAligns TopHat and STAR was calculated used as filter criterion (Max nAligns). **A:** All gap-sites (Max nAligns $>$ 0). **B:** All gap-sites (Max nAligns $>$ 1). **C:** All gap-sites (Max nAligns $>$ 10). **D:** All gap-sites (Max nAligns $>$ 100).

3 MaxEnt scores

3.1 Distribution of MaxENT scores

The range of observed MaxENT scores in TopHat and STAR alignments is shown in Table 1.

		TopHat		Star	
	Strand	Min	Max	Min	Max
score5	+	-40.8	11.8	-49.2	11.8
score5	-	-40.8	11.8	-49.2	11.8
score3	+	-59.8	15.9	-68.5	15.9
score3	-	-61.0	15.6	-69.6	15.6

Minimum and Maximum of observed MaxENT scores.

Table 1: Range of MaxENT scores

On annotated splice-sites (restricted to , MaxENT *score5*is in the range between -46.9 and 11.8 and *score3*is in the range between -57.6 and 16.1.

3.2 Usage of both scores in *wgis*

As both MaxENT scores (*score5* and *score3*) are included in *wgis*, the question arises whether only one of them would already suffice as quality criterion. However, in preliminary analysis it turned out that without usage of *score3*, *wgis*-validated gap-sites had $\approx 98\%$ GT at 5' sites but only 55 - 75 % AG at 3' sites. Thus both scores were included.

3.3 Calculation of strand orientation from MaxEnt scores

Comparison of MaxENT scores calculated in left-to-right ("+"-strand) and right-to-left (reverse complement, "-"-strand) reading direction can be used as estimate for strand orientation. A putative splice-junction with a higher score in '+' orientation than in '-' orientation will be assigned to "+"-strandand to "-"-strandotherwise.

3.3.1 Limitations of calculation model

A prerequisite for this approach is that a on direction of a gap-site receives a reasonable high score (for example '+'-direction +5 and '-'-direction -5). When MaxENT scores in both directions are low, the rating essentially states that a gap-site has low similarity with known splice-sites in either direction. As for both sores (*score5* and *score3*) a limit of 0 is used for validation of gap-sites (in *wgis*), this also seems to be a reasonable limit for usage in estimation of strand orientation.

3.3.2 Selection of score for estimation of strand orientation

Both MaxENT scores (*score5* and *score3*) potentially may be used for strand estimation. Therefore, the agreement of strand estimation from both scores was evaluated in different scenarios. On all (unfiltered) gap-sites, both scores produce different strand predictions on 64.208 (6.42 %) gap-sites for TopHat alignments and in 2.877.006 (44.35 %) gap-sites for Star alignments. On *gqs* validated gap-sites, differing strand predictions are identified on 690 (0.44 %) and 2,249 (0.035 %) in TopHat and STAR alignments respectively. On *wgis* validated gap-sites, differing strand predictions are identified on 1131 (0.15 %) and 5,598 (0.52 %) in TopHat and STAR alignments respectively.
Although, calculation of MaxENT, *score5* requires 9 nucleotides (3 exonic and 6 intronic) and *score3* requires 23 nucleotides (3 exonic and 20 intronic) only a subset of the provided sequence actually is used. Details can be viewed in the C implementation in *spliceSites* (spliceSites.c: maxent_score3 and maxent_score5 functions). For *score5*, 8 nucleotides are used and 11 for *score3*. Thus greater accuracy of one of the scores is only partially related to length of provided sequence. The *score3* was considered to be appropriate mainly because the intronic pyrimidines provide a prominent feature (besides AG).

3.4 Empirical base for MaxENT thresholds

For both MaxENT scores (*score5* and *score3*), a threshold of 1 is applied because both score counts have a local minimum nearby (Figure 4 A). Application of progressively increasing thresholds for nAligns (Figure 4 B,C,D) shows that predominantly gap-sites with MaxENT scores < 1 are filtered out.

3.5 Distribution of MaxENT scores on splice sites from minor spliceosome

In STAR alignments, 2,000 gap-sites with *AT-AC* intronic dinucleotide pairs are validated by *gqs*, but no *AT-AC* gap-site is validated by *wgis*. Thus MaxENT *score5* or *score3* scores for these gap-sites must be < 1 (the defined threshold). In order to identify small MaxENT scores, *AT-AC* gap-sites were filtered from STAR alignments and maximal *score5* and *score3* are plotted in

Figure 6. The majority of *AT-AC* gap-sites are not validated by *wgis* because *score3* values are between 0 and -10.

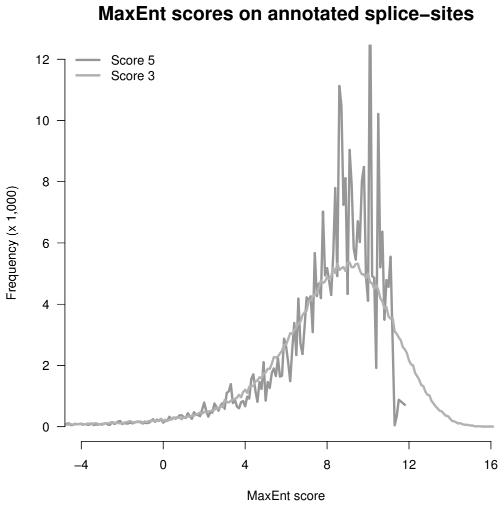

Figure 5: Distribution of MaxEnt scores on annotated splice-sites

Distribution of MaxENT scores on annotated splice-sites. 92.6 % of *score5* and *score3* values are greater than 1.

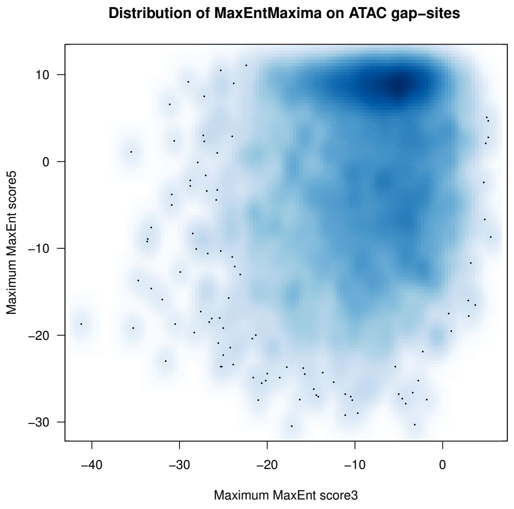

Figure 6: Distribution of maximal MaxEnt scores on "AT-AC" splice-sites from STAR alignments

Alignments from STAR aligner were filtered for "AT-AC" intronic dinucleotide pairs. MaxENT scores are calculated for each strand orientation ("+"-strand: Original sequence; "-"-strand: Reversed complement complement sequence) and the maximum of both is used. Distribution of maximum scores for *score5* and *score3* are plotted.

4 Annotated splice sites

4.1 MaxEnt scores for annotated splice-sites

In Ensemble 82, 60,448 different gene-id's and 198,455 transcript-id's are defined. Annotated splice-sites from Ensembl 82 were imported into the gap-sites analysis structures. Using this infrastructure, MaxEnt scores were calculated and splice-site sequence properties were analysed. The total number of splice-sites was 347,536. Therefrom, 175,243 are assigned to "+"-strand and 172,293 are assigned to "-"-strand. The range of MaxENT scores is shown in Table 2.

	Min	Max
score5	-46.9	11.8
score3	-57.6	16.1

Table 2: MaxENT on annotated splice-sites

On annotated splice-sites, more than 94.8 % of splice-sites are assigned MaxENTscores > 1 (the threshold for *wgis* validation) in each strand direction and for each score (*score5* and *score3*, Table 3). Altogether, 92.3 % of annotated splices sites suffice the criteria for *wgis*-validation (provided *qsm*$\geq$16).

	Strand	
	+	-
score5> 1	95.1 %	94.9 %
score3> 1	95.0 %	94.9 %

Table 3: MaxENT on annotated splice-sites

The distribution of MaxENT scores on annotated splice-sites is shown in Figure 7. Sequence logos for 5' splice-sites and 3' splice-sites are shown in Figure 8.

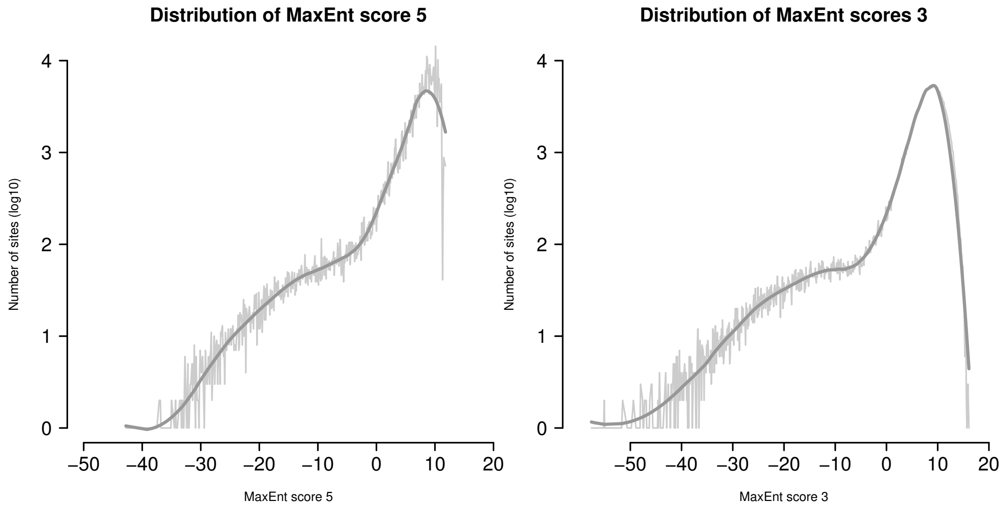

Figure 7: Distribution of MaxEnt score values

Distribution of MaxEnt scores on annotated splice-sites in Ensembl 82. A smoothed estimation line is derived from loess regression. *Left:* Distribution of 5' MaxEnt scores (score 5). *Right:* Distribution of 3' MaxEnt scores (score 3).

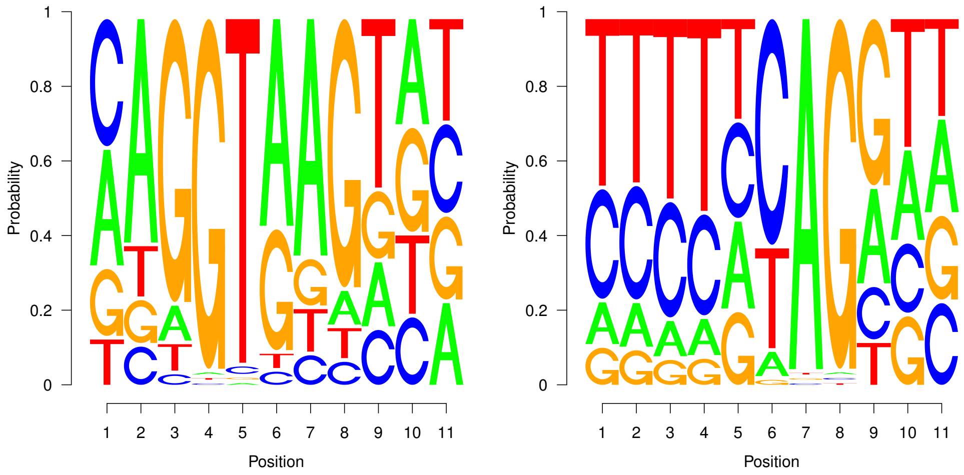

A: 5' splice-junctions **B:** 3' splice-junctions

Figure 8: Sequence logo of annotated splice-sites

Sequence logo *A (Left):* Sequence logo for 5' splice-junctions. *B (Right):* Sequence logo for 3' splice-junctions.

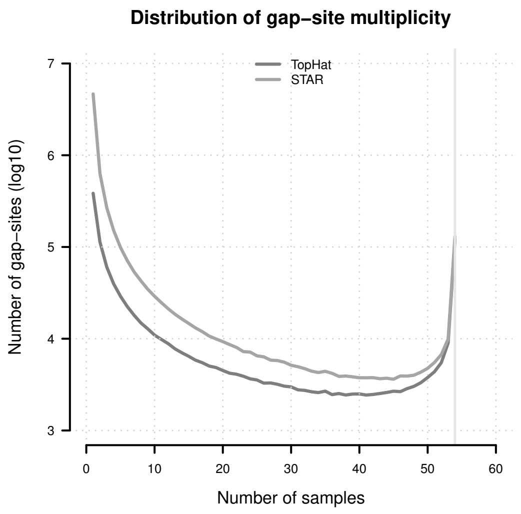

Figure 9: Proportion of samples in which gap-sites are detected.

Gap-sites counts (given in log10) observed in different numbers of samples (*nProbes*= gap-site multiplicity). The U-shaped distribution indicates that gap-sites tend to be present either in an either low or high proportion of samples.

5 Properties of *gqs* and *wgis*

IDIN and IDIN-pairs in strand-corrected orientation (5' to 3') are shown in italic letters. Non cursive printed nucleotides represent uncorrected genomic sequence (left to right).

5.1 Global sample properties

Global gap-sites statistics from alignment of 54 fibroblast transcriptomes with TopHat and STAR are summarised in Table 4.

	TopHat		Star	
nAligns=1	274	27.4 %	4,437	68.4 %
nAligns$<$10	582	58.2 %	5,939	91.6 %
nAligns$> 10^3$	136	13.6 %	121	1.9 %
nProbes=1	385	38.4 %	4,655	71.8 %
nProbes=2	113	11.3 %	632	9.7 %
nProbes=54	120	12.0 %	130	2.0 %
sod=0	244	24.3 %	256	3.9 %
gqs=1000	156	18.5 %	166	2.6 %
wgis$\neq$ 0	770	77.0 %	1,066	16.4 %

Absolute number of gap-sites $\times$ 1,000.

Table 4: Global distribution of gap-sites

5.2 Comparison of *gqs* and *wgis* validation

In general, STAR reports less *gqs* and *wgis*-validated gap-sites with nAligns $>$ 10,000 and more *gqs* and *wgis*-validated gap-sites with nAligns $<$ 100 (Figure 11).

5.2.1 *gqs* and *wgis* validation of high abundant gap-sites

In TopHat alignments, 91.7 % of gap-sites with nAligns $>$ 9,526 (the 95% quantile) are validated by *gqs*. In STAR alignments, 95.0 % of gap-sites with nAligns $>$ 5,601 (the 99% quantile) are validated by *gqs*. Missing validation by *gqs* in these cases is in the vast majority (97.0 % in TopHat, 99.4 % in STAR) due to *qsm*$<$ 200.
In TopHat alignments, 79.2 % of gap-sites with nAligns $>$ 9,526 (the 95% quantile) are validated by *wgis*. In STAR alignments, 19.2 % of gap-sites with nAligns $>$ 5,601 (the 99% quantile) are validated by *wgis*.

5.2.2 *gqs* and *wgis* validation of low abundant gap-sites

From TopHat alignments, 37,909 gap-sites with less than 1,000 nAligns are validated by *gqs*. STAR reports 52,804 *gqs*-validated gap-sites within this range, 39.3 % more.
From TopHat alignments, 642,396 gap-sites with less than 1,000 nAligns are validated by *gqs*. In STAR alignments 947,623 gap-sites with nAligns $<$ 1,000 are present, 47.5 % more than in TopHat alignments.

5.2.3 Alternative validation limits for *gqs*

Alternatively to annotation based validation limit (median(sod) $\approx$ 0) the proportion of *GT-AG*-sites can be utilised for determination of validation limits for *gqs*. The proportion of *GT-AG*-sites can be estimated by adding the proportion of GT-AG-sites and CT-AC-sites. When for example *gqs*-validated gap-sites shall contain > 90 % *GT-AG*-sites, *gqs*-limits of 340 in TopHat alignments and 940 in STAR alignments are required (Figure 10).

5.2.4 Proportion of samples in which gap-sites are identified

The proportion of samples in which a gap-site is identified (gap-site proportion) can be used as qualifying criterion for gap-sites when the number of samples is large enough. The exact distribution of gap-site proportions in relation to both gap-site scores (*gqs* and *wgis*) is shown in Figure 13.

5.2.5 Gap-sites present in all samples

The distribution of |*wgis*| values in gap-sites present in all samples is shown in Figure 14. In TopHat alignments, 2,941 gap-sites are not *wgis*-validated but present in all 54 samples. In alignments from STAR, 2,043 gap-sites are assigned *wgis*= 0 and are identified in all samples.

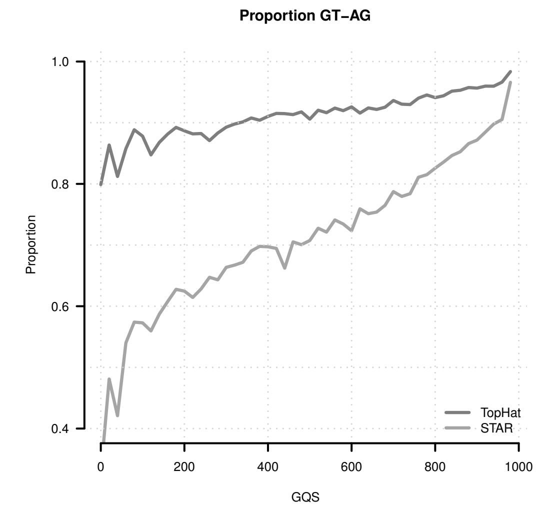

Figure 10: Proportion of GT-AG-sites for different GQS levels.

Gap-sites were categorised by *gqs* (using intervals of length 20 as categories. Proportion of *GT-AG* sites is estimated by adding proportion of GT-AG and CT-AC sites in each *gqs* category.

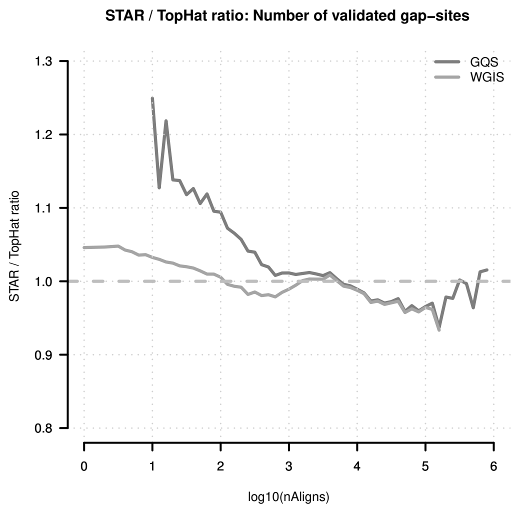

Figure 11: STAR / TopHat ratio of number of validated gap-sites

Gap-sites were categorised according to number of supporting alignments (nAligns): The log10(nAligns) value rounded to one digit was used as category (x-axis). Absolute numbers of validated gap-sites validated by *gqs* and *wgis* are compared between STAR and TopHat alignments. Lines show STAR / TopHat ratio for *gqs* and *wgis*.

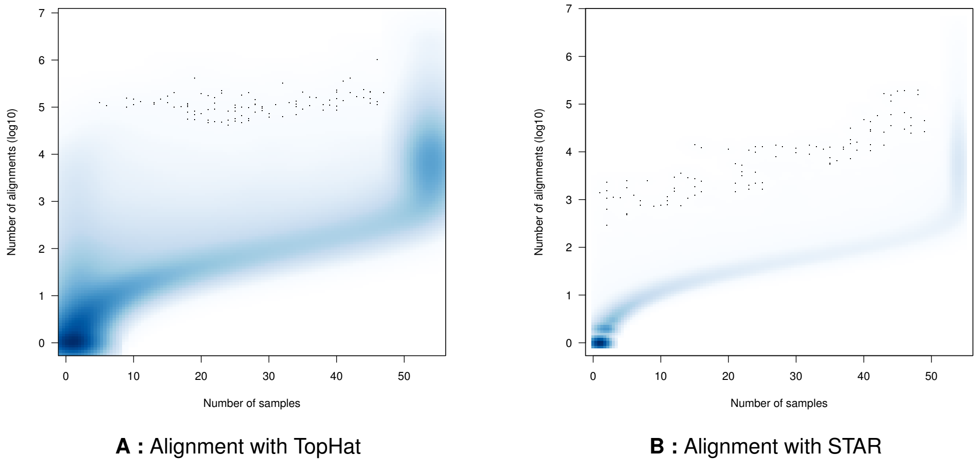

Figure 12: Number of supporting alignments

Tabled supporting alignments (given in log10) in relation to number of samples in which gap-sites are present **A (Left):** Alignment from TopHat aligner. **B (Right):** Alignment from STAR aligner.

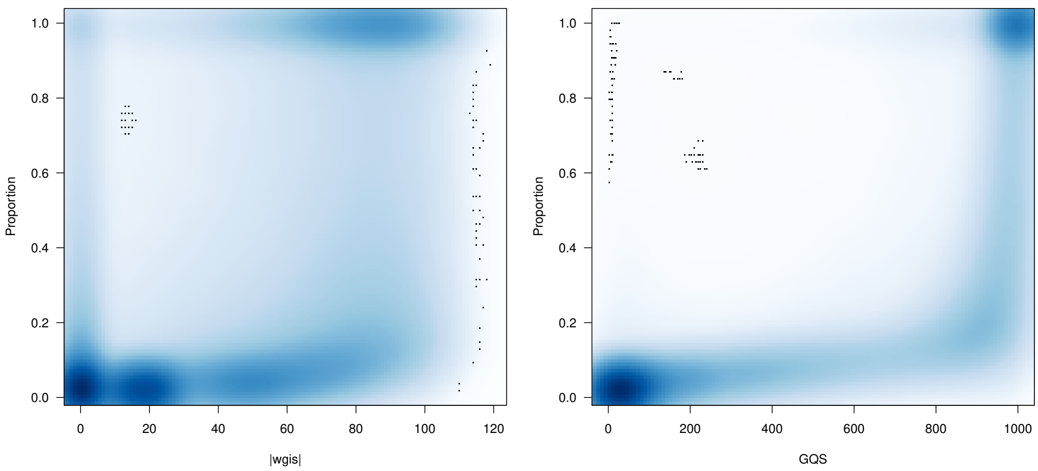

Figure 13: Density estimates for gap-site proportions and *gqs* and *wgis*

Proportion of samples denotes the proportion of samples in which a gap-site is identified (calculated as nProbes / 54). **Left:** Proportions for different *gqs* values. **Right:** Proportions for different $|wgis|$ values.

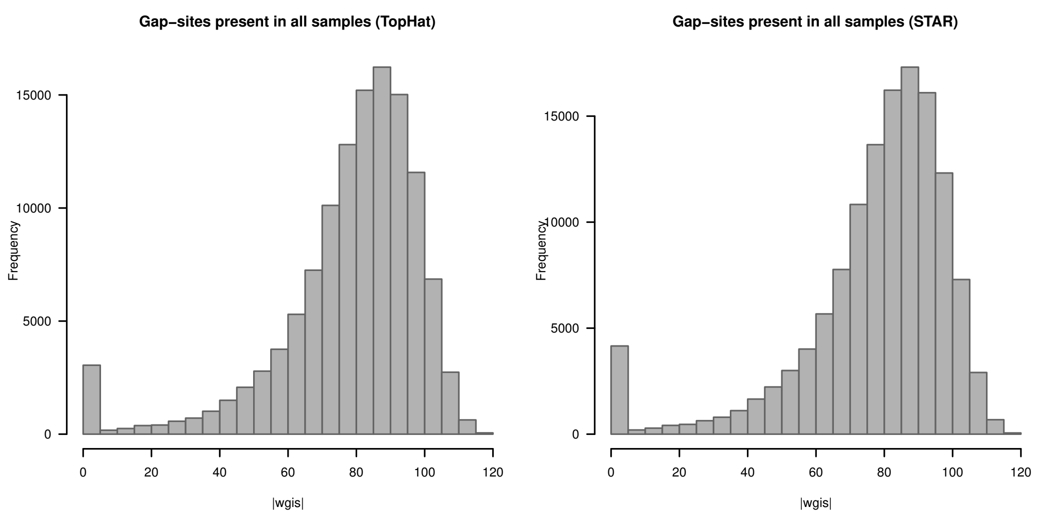

Figure 14: Histogram of |*wgis*| values for gap-sites present in all (54) samples

Left: Frequency in alignments from TopHat. **Right:** Frequency in alignments from STAR.

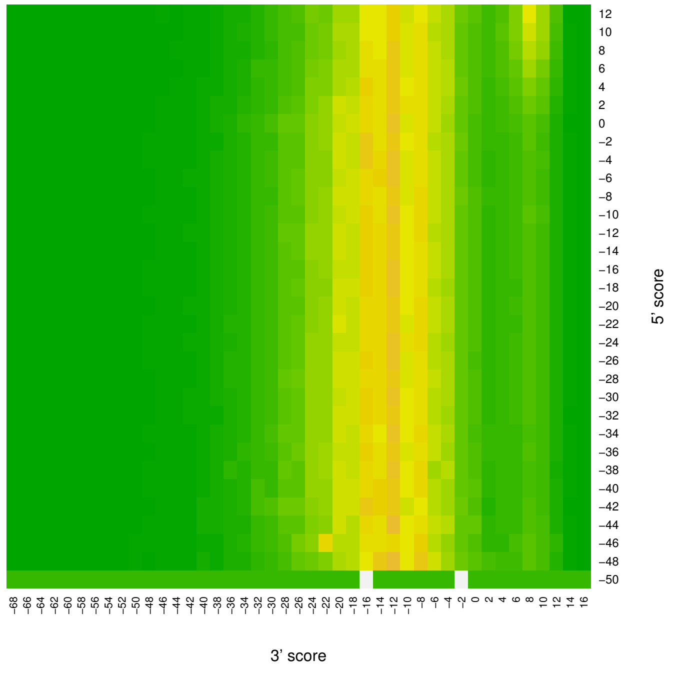

Figure 15: Heatmap of MaxEnt score5 and score3 values

Heatmap of *score5* and *score3* values indicating that simultaneous truncation of *score5* and *score3* at values of 1 separates two local maxima from each other in the plane.

5.3 Analysis for GQL levels

5.3.1 Sequence logos

Sequence logos for exon-intron boundaries from STAR alignments for all *gql*-levels are shown in Figure 16.

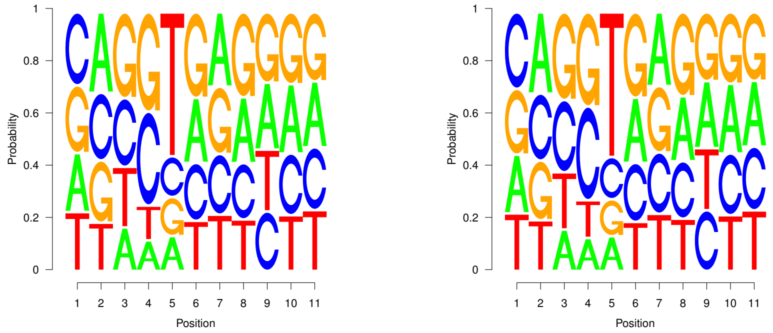

A : All gap-sites (left side, L->R direction)

B : All gap-sites (right side R-> direction)

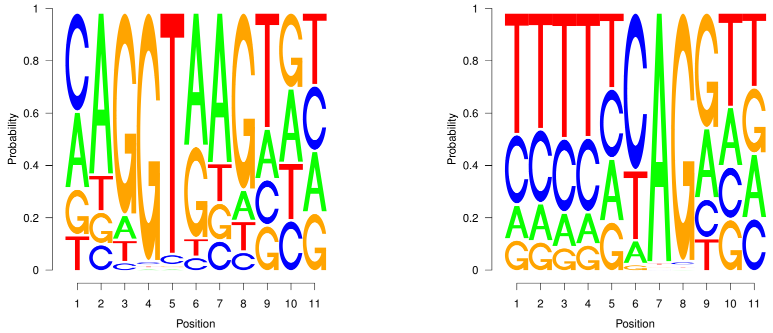

C : GQL 1 gap-sites (5' direction)

D : GQL 1 gap-sites (3' direction)

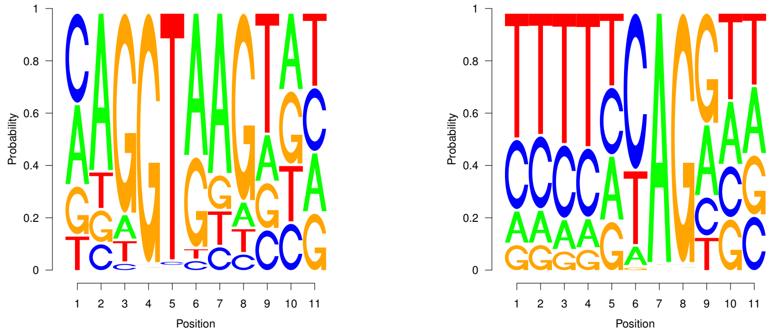

E : GQL 2 gap-sites (5' direction)

F : GQL 2 gap-sites (3' direction)

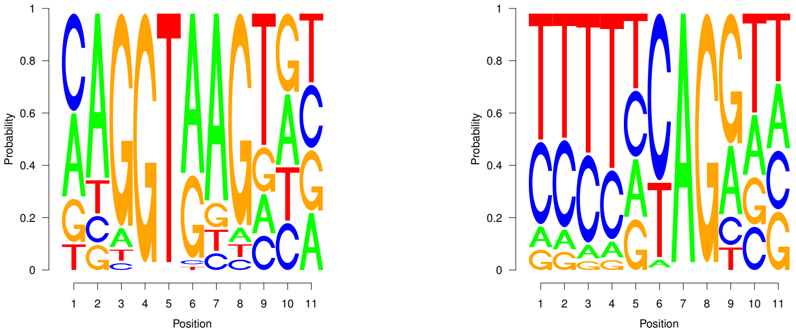

G : GQL 3 gap-sites (5' direction)

H : GQL 3 gap-sites (3' direction)

Figure 16: Sequence logos for exon-intron boundaries from STAR alignments

6 Total number of splicing events in human tissues

In order to provide a realistic magnitude for the total number of splicing events present in human samples, some external data sources are consulted. A first human global analysis of splice sites reported 43,337 splice junctions [1]. In data downloaded from H-DBAS [2] (Human-transcriptome Database for Alternative Splicing) we identified 157.162 unique junctions (using R package *refGenome*). A survey on splicing in immune cells (from the Immunological Genome Project) describes 92,562 splice-junctions in CD4$^+$ T-cells and 91,882 splice-junctions in CD19$^+$ B-cells [3]. In a recent survey of splice-sites in human transcriptome using RNA-seq data from 16 human tissues approximately 220,000 splice sites were detected [6]. ENCODE identified 41,204 genes, 73,325 transcripts, 94,800 exons and 69,052 splice-junctions using Cufflinks [2]. In the analysis of human transcriptome published by GTEx, in total 329,984 splice junctions were reported [5] Analysis of Ensembl 82 (using CRAN *refGenome*) resulted in 60,448 different gene-id's and 198,455 transcript-id's. The total number of unique splice-sites was 347,536.

Taken together, the observed total number of splice-sites in cells from a human tissue are in the range between 50,000 and 500,000. Thus, gap-site numbers exceeding this range presumably contain a considerable fraction of artificial results which do not represent biological events.

[2] http://h-invitational.jp/h-dbas/

7 Gap-sites with high alignment coverage

For a certain cell type, the collection of maximal expressed genes can be exprected to reflect the physiological role of associated tissues. Gap-sites with maximal alignment coverages in the analyzed fibroblast transcriptomes are collected in Table 5. Fibroblast markers, as Collagen 1α2 and Vimentin are present, while Platelet Derived Growth Factor (PDGF1) is missing.

Name	Sites	Name	Sites	Name	Sites	Name	Sites	Name	Sites
COL1A1	48	CTSK	7	TPT1	5	FTL	3	TMSB10	2
COL1A2	45	SERPINE2	7	THBS1	4	LGALS1	3	TMSB4X	2
FN1	44	COL6A1	6	ACTB	3	ACTG1	2	CD63	1
COL6A2	19	EEF1A1	6	B2M	3	CTSB	2	SPARC	1
DCN	8	GAPDH	6	COL3A1	3	RPS18	2	TIMP1	1
VIM	8	MMP2	5	FTH1	3	S100A6	2		

Table 5: Genes containing gap-sites with high alignment coverage from STAR alignments. Gap-sites with high number of alignment coverage (nAligns $> 10^6$) were extracted (in total 251 sites). All gap-sites are located on annotated splice-sites (*sod*=0). The number of gap-sites annotated for each gene and gene name is displayed.

8 Miscellaneous remarks

8.1 Logarithm for base *a*

The relation of the logarithm for base *a* and the natural logarithm can be obtained with a few simple calculations: Thus, logarithm functions for different bases only differ by a constant factor.

$$a^x = (e^{\log(a)})^x = e^{x\log(a)} \quad (1)$$

$$\Rightarrow x = a^{\log_a(x)} = e^{\log_a(x)\log(a)} \quad (2)$$

$$\Rightarrow \log(x) = \log_a(x)\ \log(a) \quad (3)$$

References

[1] M. Burset, I. A. Seledtsov, and V. V. Solovyev. Analysis of canonical and non-canonical splice sites in mammalian genomes. *Nucleic Acids Res.*, 28(21):4364–4375, Nov 2000.

[2] S. Djebali, C. A. Davis, A. Merkel, A. Dobin, T. Lassmann, A. Mortazavi, A. Tanzer, J. Lagarde, W. Lin, F. Schlesinger, C. Xue, G. K. Marinov, J. Khatun, B. A. Williams, C. Zaleski, J. Rozowsky, M. Roder, F. Kokocinski, R. F. Abdelhamid, T. Alioto, I. Antoshechkin, M. T. Baer, N. S. Bar, P. Batut, K. Bell, I. Bell, S. Chakrabortty, X. Chen, J. Chrast, J. Curado, T. Derrien, J. Drenkow, E. Dumais, J. Dumais, R. Duttagupta, E. Falconnet, M. Fastuca, K. Fejes-Toth, P. Ferreira, S. Foissac, M. J. Fullwood, H. Gao, D. Gonzalez, A. Gordon, H. Gunawardena, C. Howald, S. Jha, R. Johnson, P. Kapranov, B. King, C. Kingswood, O. J. Luo, E. Park, K. Persaud, J. B. Preall, P. Ribeca, B. Risk, D. Robyr, M. Sammeth, L. Schaffer, L. H. See, A. Shahab, J. Skancke, A. M. Suzuki, H. Takahashi, H. Tilgner, D. Trout, N. Walters, H. Wang, J. Wrobel, Y. Yu, X. Ruan, Y. Hayashizaki, J. Harrow, M. Gerstein, T. Hubbard, A. Reymond, S. E. Antonarakis, G. Hannon, M. C. Giddings, Y. Ruan, B. Wold, P. Carninci, R. Guigo, and T. R. Gingeras. Landscape of transcription in human cells. *Nature*, 489(7414):101–108, Sep 2012.

[3] A. Ergun, G. Doran, J. C. Costello, H. H. Paik, J. J. Collins, D. Mathis, C. Benoist, D. A. Blair, M. L. Dustin, S. A. Shinton, R. R. Hardy, T. Shay, A. Regevc, N. Cohen, P. Brennan, M. Brenner, F. Kim, T. Nageswara Rao, A. Wagers, T. Heng, J. Ericson, K. Rothamel, A. Ortiz-Lopez, D. Mathis, C. Benoist, T. Kreslavsky, A. Fletcher, K. Elpek, A. Bellemare-Pelletier, D. Malhotra, S. Turley, J. Miller, B. Brown, M. Merad, E. L. Gautier, C. Jakubzick, G. J. Randolph, P. Monach, A. J. Best, J. Knell, A. Goldrath, V. Jojic, D. Koller, D. Laidlaw, J. Collins, R. Gazit, D. J. Rossi, N. Malhotra, K. Sylvia, J. Kang, N. A. Bezman, J. C. Sun, G. Min-Oo, C. C. Kim, and L. L. Lanier. Differential splicing across immune system lineages. *Proc. Natl. Acad. Sci. U.S.A.*, 110(35):14324–14329, Aug 2013.

[4] J. E. Hooper. A survey of software for genome-wide discovery of differential splicing in RNA-Seq data. *Hum. Genomics*, 8:3, Jan 2014.

[5] M. Mele, P. G. Ferreira, F. Reverter, D. S. DeLuca, J. Monlong, M. Sammeth, T. R. Young, J. M. Goldmann, D. D. Pervouchine, T. J. Sullivan, R. Johnson, A. V. Segre, S. Djebali, A. Niarchou, F. A. Wright, T. Lappalainen, M. Calvo, G. Getz, E. T. Dermitzakis, K. G. Ardlie, and R. Guigo. Human genomics. The human transcriptome across tissues and individuals. *Science*, 348(6235):660–665, May 2015.

[6] G. E. Parada, R. Munita, C. A. Cerda, and K. Gysling. A comprehensive survey of non-canonical splice sites in the human transcriptome. *Nucleic Acids Res.*, 42(16):10564–10578, 2014.
